# Patterning of Leaf Vein Networks by Convergent Auxin Transport Pathways

**DOI:** 10.1371/journal.pgen.1003294

**Published:** 2013-02-21

**Authors:** Megan G. Sawchuk, Alexander Edgar, Enrico Scarpella

**Affiliations:** Department of Biological Sciences, University of Alberta, Edmonton, Alberta, Canada; Peking University, China

## Abstract

The formation of leaf vein patterns has fascinated biologists for centuries. Transport of the plant signal auxin has long been implicated in vein patterning, but molecular details have remained unclear. Varied evidence suggests a central role for the plasma-membrane (PM)-localized PIN-FORMED1 (PIN1) intercellular auxin transporter of *Arabidopsis thaliana* in auxin-transport-dependent vein patterning. However, in contrast to the severe vein-pattern defects induced by auxin transport inhibitors, *pin1* mutant leaves have only mild vein-pattern defects. These defects have been interpreted as evidence of redundancy between PIN1 and the other four PM-localized PIN proteins in vein patterning, redundancy that underlies many developmental processes. By contrast, we show here that vein patterning in the Arabidopsis leaf is controlled by two distinct and convergent auxin-transport pathways: intercellular auxin transport mediated by PM-localized PIN1 and intracellular auxin transport mediated by the evolutionarily older, endoplasmic-reticulum-localized PIN6, PIN8, and PIN5. PIN6 and PIN8 are expressed, as PIN1 and PIN5, at sites of vein formation. *pin6* synthetically enhances *pin1* vein-pattern defects, and *pin8* quantitatively enhances *pin1pin6* vein-pattern defects. Function of *PIN6* is necessary, redundantly with that of *PIN8*, and sufficient to control auxin response levels, PIN1 expression, and vein network formation; and the vein pattern defects induced by ectopic *PIN6* expression are mimicked by ectopic *PIN8* expression. Finally, vein patterning functions of *PIN6* and *PIN8* are antagonized by *PIN5* function. Our data define a new level of control of vein patterning, one with repercussions on other patterning processes in the plant, and suggest a mechanism to select cell files specialized for vascular function that predates evolution of PM-localized PIN proteins.

## Author Summary

The beautiful and varied patterns of veins in plant leaves have intrigued both artists and scientists since antiquity. The seminal work of some of these scientists has shown that the plant hormone auxin and its transport in plant tissues control leaf vein patterning, but molecular details of auxin-transport-dependent vein patterning are still unknown. Here we find that vein patterning is controlled by the concerted action of two spatially separate auxin transport pathways. One pathway transports auxin from cell to cell, the other to and from different compartments within the cell. The cell-to-cell pathway of auxin transport seems to exist only in land plants with veins. The within-cell pathway of auxin transport seems to have appeared earlier in the evolution of plants and exists also in primitive land plants, which form vein-like structures. Our findings suggest an unsuspected level of control of vein patterning, one that regulates patterning of plant features beyond veins and that could even be relevant for the formation of vein-like structures of primitive land plants.

## Introduction

Branched structures pervade all levels of organization in living organisms, from molecules to organelles, cells, multicellular organs and entire organisms, and the principles that guide the formation of these branched structures have long been object of interest of biologists and mathematicians. However, few branched structures have historically captured more widespread attention than the vein networks of plant leaves. From a developmental standpoint, such attention seems justified as files of vein precursor cells are selected from within a population of seemingly identical cells [1], [2]. Furthermore, in most species the product of this patterning process is both reproducible and variable: reproducible as vein networks supply all areas of the leaf; variable as the exact sites of vein formation are unpredictable [3]. These observations argue against rigid specification of leaf vein patterns and suggest a self-organizing control mechanism that reconciles the plasticity of vein formation with the stringent requirement for organ vascularization [4].

Though the identity of the molecules involved is largely unknown, varied evidence supports a decisive role for the polar, cell-to-cell transport of the plant signal auxin in leaf vein patterning: auxin application induces formation of new veins oriented towards pre-existing veins [3]; the inductive and orienting effect of applied auxin on vein formation is suppressed by auxin transport inhibitors [5]; and auxin transport inhibitors induce characteristic defects in vein patterns [6], [7]. During leaf development, expression of the plasma-membrane (PM)-localized PIN-FORMED1 (PIN1) auxin transporter of *Arabidopsis thaliana* [8], [9] is initiated in broad tissue domains and becomes gradually restricted to single files of vascular precursor cells [10], [11]. Narrowing of PIN1 expression domains is associated with polarization of PIN1 subcellular localization to the presumed auxin-efflux side of PIN1-expressing cells. Auxin levels define the initial size of PIN1 expression domains, and both domain narrowing and PIN1 polarization are obstructed by auxin transport inhibitors. These observations agree with conceptual formulation [12], and mathematical modeling (reviewed in [14]–[18]) of progressive restriction of nondirectional auxin transport across tissues to polar transport in single files of vascular precursor cells by positive feedback between auxin flow through a cell and the cell's auxin conductivity. However, in contrast to the severe vein-pattern defects induced by auxin transport inhibitors [6], [7], vein pattern defects in *pin1* mutant leaves are mild [6], [19], [20]. PIN1 is member of a family comprising four other PM-localized PIN proteins and three, evolutionary older, endoplasmic reticulum (ER)-localized PIN proteins [21]–[24]. Thus, the mild vein-pattern defects of *pin1* have been attributed to redundancy among PM-localized PIN proteins (e.g., [6], [10], [19], [25]), redundancy that has been shown to underlie, to different extents, many other developmental processes ([20], [26], [27] and references therein).

Here we show that the vein network of the Arabidopsis leaf is patterned by two distinct and convergent auxin transport pathways: intercellular auxin transport mediated by PM-localized PIN1 and intracellular auxin transport mediated by ER-localized PIN6, PIN8 and PIN5. Our results suggest an ancestral auxin-transport-dependent mechanism to specify cell files to vascular function that predates evolution of PM-localized PIN proteins.

## Results

### Vein patterning functions of Arabidopsis *PIN* genes

**Figure 1:**
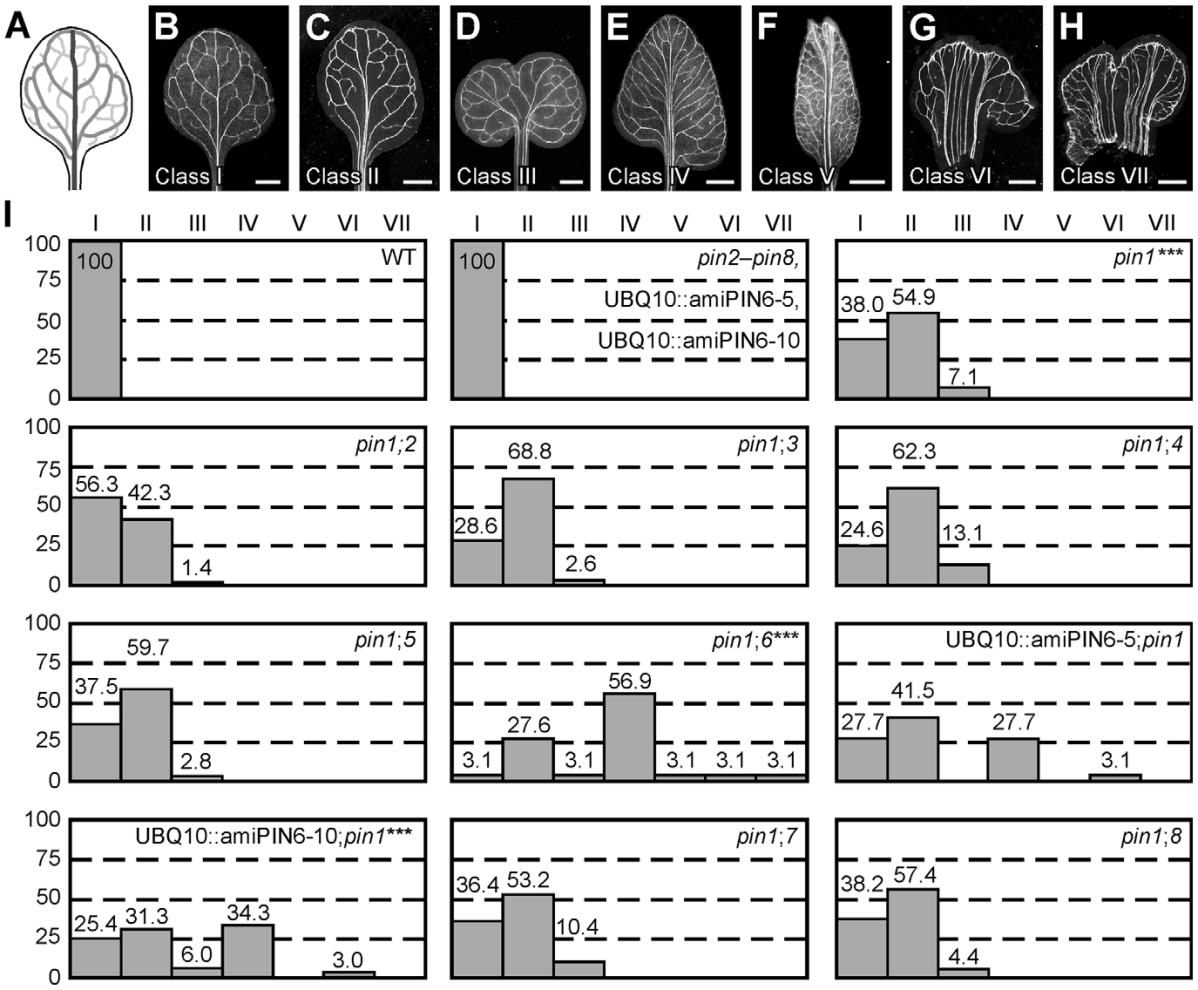
Vein patterning functions of Arabidopsis *PIN* genes. (A,B) Vein pattern of WT mature first leaf. In (A), dark grey, midvein; grey, loops; light grey, minor veins. (B–H) Dark-field illumination of cleared mature first leaves illustrating phenotype classes: unbranched, narrow midvein and scalloped vein-network outline (B); bifurcated midvein and scalloped vein-network outline (C); fused leaves with scalloped vein-network outline (D); conspicuous marginal vein (E); fused leaves with conspicuous marginal vein (F); wide midvein (G); fused leaves with wide midvein (H). (I) Percentages of leaves in phenotype classes. Difference between *pin1* and WT, between *pin1*;*6* and *pin1*, and between UBQ10::amiPIN6;*pin1* and *pin1* was significant at *P*<0.001 (***) by Kruskal-Wallis and Mann-Whitney test with Bonferroni correction. Sample population sizes: WT, 65; *pin2*, 68; *pin3*, 68; *pin4*, 68; *pin5*, 68; *pin6*, 68; UBQ10::amiPIN6-5, 65; UBQ10::amiPIN6-10, 65; *pin7*, 68; *pin8*, 68; *pin1*, 71; *pin1*;*2*, 71; *pin1*;*3*, 77; *pin1*;*4*, 69; *pin1*;*5*, 72; *pin1*;*6*, 65; UBQ10::amiPIN6-5;*pin1*, 65; UBQ10::amiPIN6-10;*pin1*, 67; *pin1*;*7*, 77; *pin1*;*8*, 68. Bars: (B–F) 1.5 mm; (G,H) 0.75 mm.

WT Arabidopsis grown under normal conditions forms separate leaves, whose vein patterns are defined by reproducible features [28], [29] (Figure 1A and 1B): a narrow, central midvein that runs the length of the leaf; lateral veins that branch from the midvein and join distal veins to form closed loops; minor veins that branch from midvein and loops and either end freely or join other veins; and minor veins and loops that curve near the leaf margin, lending a scalloped outline to the vein network. By contrast, WT leaves developed in the presence of auxin transport inhibitors often show separation defects (‘fused leaves’), and vein patterns of auxin-transport-inhibited leaves deviate from the norm in at least four respects [6], [7]. First, the midvein bifurcates near the leaf tip. Second, the vein network consists of more lateral veins. Third, lateral veins do not join the midvein at the centre of the leaf but run parallel to one another to form a wider midvein. Fourth, lateral veins end in a marginal vein that closely parallels the leaf margin, lending a smooth outline to the vein network. Mutation of *PIN1* (AT1G73590) approximates these defects, but vein patterns are only mildly affected in *pin1* [6], [19] (Figure 1). As *PIN1* is one of eight *PIN* genes [21]–[24] and other gene families have been implicated in auxin transport (e.g., [30]–[32]), the mild vein-pattern defects of *pin1* might reflect contribution of other auxin transporters to vein patterning; here we tested whether other *PIN* genes contribute to vein patterning.

We first explored this possibility by analyzing vein patterns of single mutants in *PIN2* (AT5G57090), *PIN3* (AT1G70940), *PIN4* (AT2G01420), *PIN5* (AT5G16530), *PIN6* (AT1G77110), *PIN7* (AT1G23080) and *PIN8* (AT5G15100). *pin2*–*pin8* (Table S1) had WT vein patterns (Figure 1), limiting nonredundant vein-patterning functions to *PIN1*. Thus, to uncover potential vein-patterning functions of *PIN2*–*PIN8*, we next analyzed vein patterns of double mutants between *pin1* and *pin2*–*pin8*. Only mutation of the auxin-transporter-encoding *PIN6* [9] had a significant effect on *pin1* phenotype spectrum, with ∼65% of *pin1pin6* (*pin1*;*6*) leaves belonging to new, stronger classes (Figure 1). *pin6* had a similar effect on *pin1* phenotype spectrum in seedlings (Figure S1): ∼95% penetrance of cotyledon defects in *pin1*;*6* vs. ∼35% in *pin1*, and appearance in *pin1*;*6* of a cup-shaped cotyledon phenotype resembling that of embryos developed in the presence of auxin transport inhibitors [33], [34]. In both leaves and seedlings, the *pin1*;*6* phenotype spectrum was mimicked by expressing an artificial microRNA targeting *PIN6* (UBQ10::amiPIN6) in the *pin1* background (Figure 1 and Figure S1). We conclude that *PIN6* is the *PIN* gene that most contributes to auxin-transport-dependent vein patterning in the absence of *PIN1* function.

### PIN6 expression in leaf development

**Figure 2:**
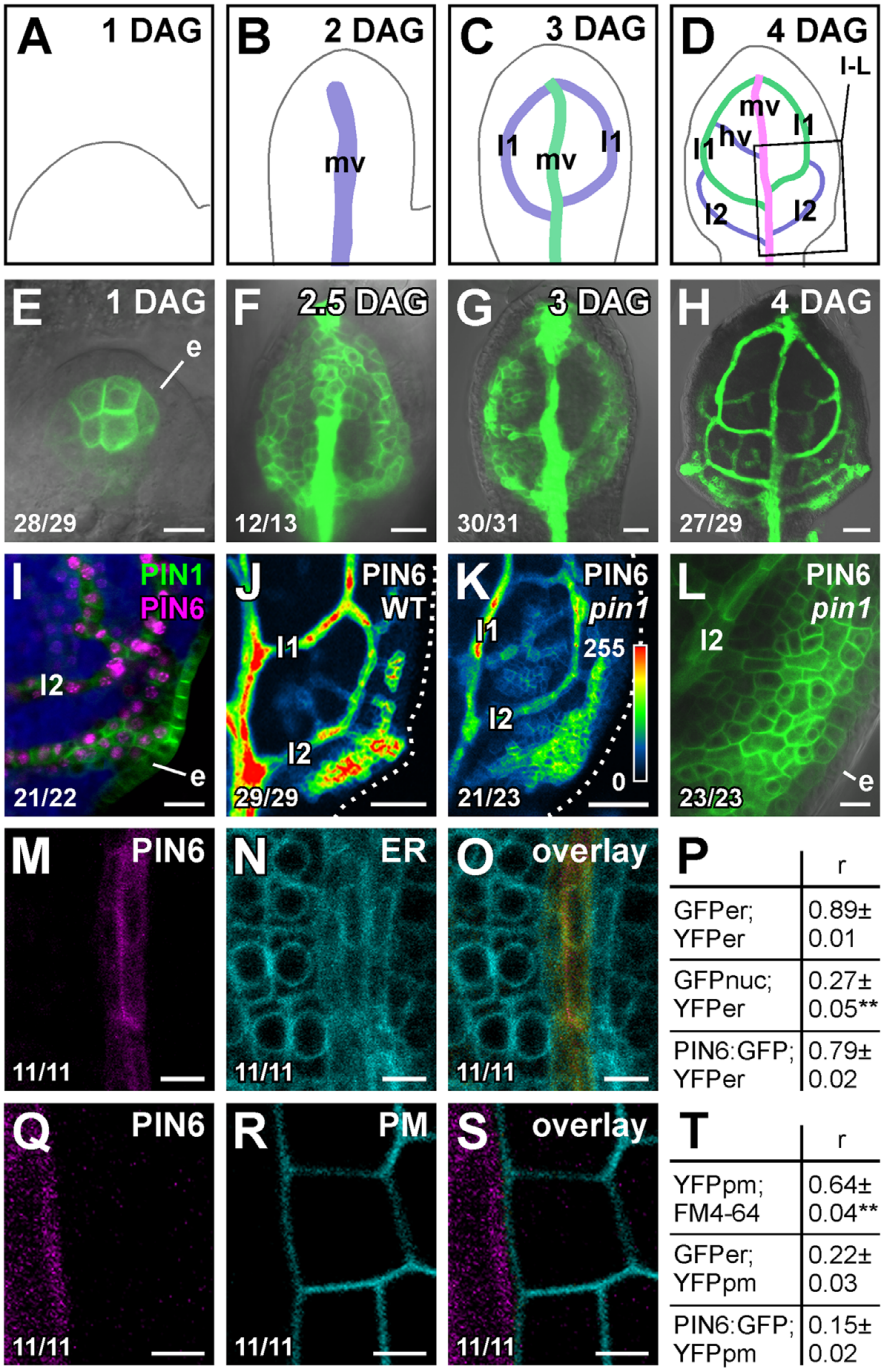
PIN6 expression in leaf development. (A–O,Q–S) Top right: leaf age in days after germination (DAG), marker and genotype. Bottom left: reproducibility index. First leaves. (A–D) Midvein, loops and minor veins differentiate progressively later in the same region of the developing leaf, and loops and minor veins differentiate in a tip-to-base sequence during leaf development [6], [7], [57]; purple, green and magenta: successive stages of vein differentiation. Box in (D) illustrates position of close-ups in (I–L). (E–O,Q–S) Confocal laser scanning microscopy with (E–H,L) or without (I–K,M–O,Q–S) transmitted light. (E–H) PIN6::PIN6:GFP expression. (I) Expression of PIN6::YFPnuc and PIN1:PIN1:CFP at 4 DAG; blue: chlorophyll. (J–L) PIN6::PIN6:GFP expression in WT (J) and *pin1* (K,L) at 4 DAG. LUT (in K) visualizes expression levels. Dotted line: leaf outline. (M–O,Q–S) Expression of PIN6::PIN6:GFP (M,Q), 35S::YFPer (N), 35S::LTI6B:YFP (R) and respective overlays displayed with a dual-channel LUT [66] (O,S): prevalence of cyan over colocalized magenta signal is shown in green, opposite in red, and colocalized cyan and magenta signals of equal intensity in yellow. (P,T) Quantification of colocalized GFP and YFP signals (as mean ± SE of Manders' coefficient ‘r’) in populations (*n* = 11) of positive controls: J1721::GFPer;35S::YFPer (P), 35S::YFPpm;FM4-64 (T); negative controls: ATHB8::GFPnuc;35S::YFPer (P), J1721::GFPer; 35S::YFPpm (T); and samples: PIN6::PIN6:GFP;35S::YFPer (P), PIN6::PIN6:GFP; 35S::YFPpm (T). (P) Difference between negative control and positive control, and between negative control and sample was significant at *P*<0.01 (**) by one-way ANOVA and Tukey's test. (T) Difference between positive control and negative control, and between positive control and sample was significant at *P*<0.01 (**) by one-way ANOVA and Tukey's test. e, epidermis; hv, minor vein; l1, first loop; l2, second loop; mv, midvein. Bars: (E,I,L) 10 µm; (F,G) 20 µm; (H,J,K) 50 µm; (M–O) 5 µm; (Q–S) 2.5 µm.

Double-mutant analysis suggests functions for *PIN6* in vein patterning (Figure 1; see Discussion). We thus asked whether *PIN6* was expressed during vein formation. To address this question, we imaged expression of a functional (Table S1) translational fusion of *PIN6* to GFP driven by the *PIN6* promoter (PIN6::PIN6:GFP). During leaf development, PIN6::PIN6:GFP expression was initiated in broad subepidermal domains that narrowed to sites of vein formation (Figure 2A–2H), suggesting PIN6 expression during vein formation. Expression of PIN6::PIN6:GFP was recapitulated by expression of a PIN6::YFPnuc transcriptional fusion (*PIN6* promoter driving expression of a nuclear YFP) (Figure S2A–S2E), which overlapped with expression of PIN1::PIN1:CFP [35] in leaf subepidermal tissues (Figure 2I). In *pin1*, PIN6::PIN6:GFP expression remained confined to subepidermal tissues, but expression was weaker (Figure 2J–2L).

A *PIN6* translational fusion localizes to the ER in tobacco suspension cells [36]. To determine PIN6 subcellular localization in Arabidopsis leaves, we quantified degree of colocalization between expression of PIN6::PIN6:GFP and expression of ER, or PM, markers. Expression of PIN6::PIN6:GFP correlated with expression of ER, but not PM, markers (Figure 2M–2T and Figure S3), suggesting ER-localization of PIN6 in vein development.

### Genetic interaction between *PIN1*, *PIN5*, *PIN6*, and *PIN8* in vein patterning

**Figure 3:**
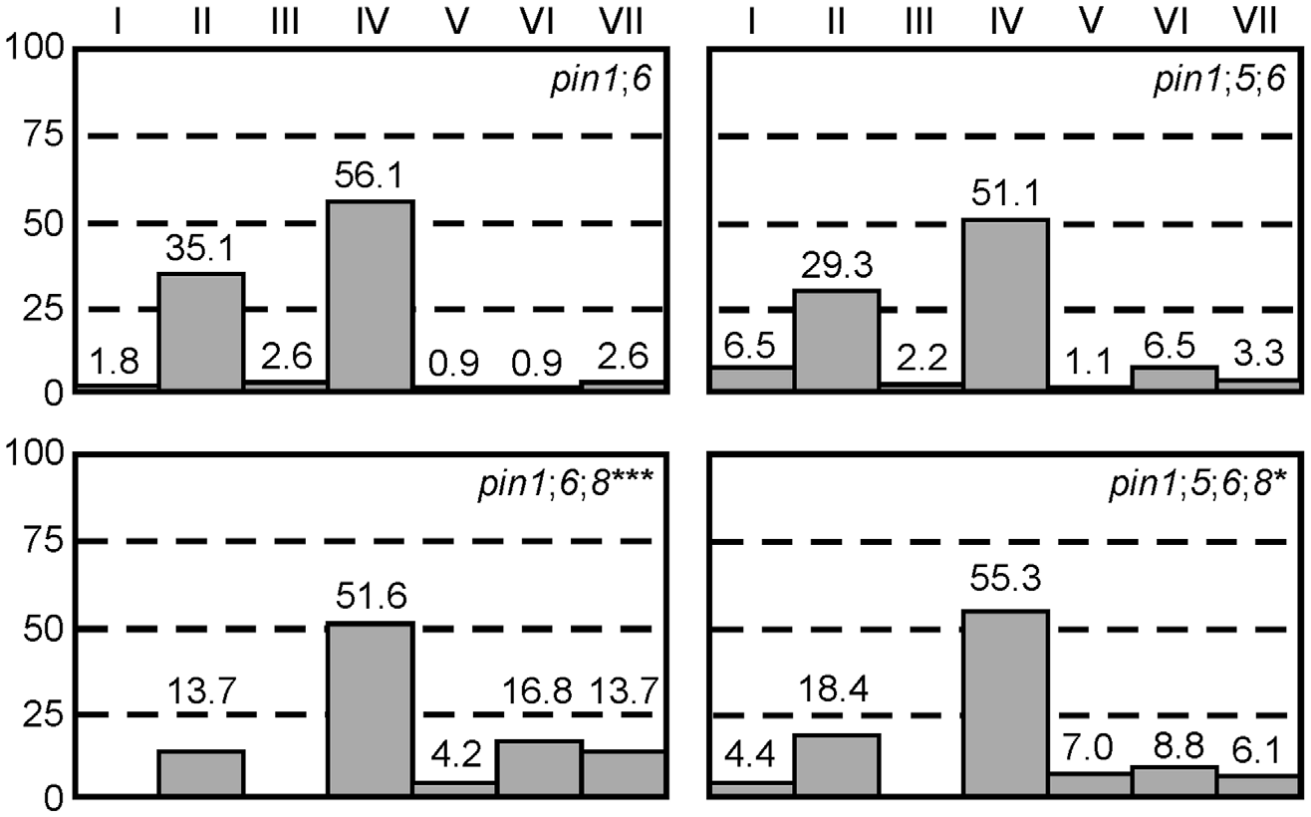
Genetic interaction between *PIN1*, *PIN5*, *PIN6*, and *PIN8* in vein patterning. Percentages of leaves in phenotype classes (defined in Figure 1). Difference between *pin1*;*6*;*8* and *pin1*;*6*, and between *pin1*;*5*;*6*;*8* and *pin1*;*6*;*8* was significant at *P*<0.05 (*) or *P*<0.001 (***) by Kruskal-Wallis and Mann-Whitney test with Bonferroni correction. Sample population sizes: *pin1*;*6*, 114; *pin1*;*5*;*6*, 92; *pin1*;*6*;*8*, 95; *pin1*;*5*;*6*;*8*, 114.

PIN6 belongs to a subfamily of proteins that includes the ER-localized PIN5 and PIN8 auxin transporters [36]–[39]. Thus, we asked whether frequencies of vein pattern phenotypes in *pin1*;*6* were affected by additional mutation of *PIN5* or *PIN8*. While *pin8* shifted the distribution of *pin1*;*6* phenotypes towards stronger classes, *pin5* partially normalized *pin1*;*6*;*8* phenotype spectrum (Figure 3A). *pin5* and *pin8* had similar effects in seedlings (Figure S1): (1) complete penetrance of cotyledon defects in *pin1*;*6*;*8* vs. ∼95% in *pin1*;*6*; (2) ∼95% penetrance of the cup-shaped cotyledon phenotype in *pin1*;*6*;*8* vs. ∼60% in *pin1*;*6*; and (3) partial normalization of cotyledon defects of *pin1*;*6*;*8* by *pin5*.

### PIN8 expression in leaf development

**Figure 4:**
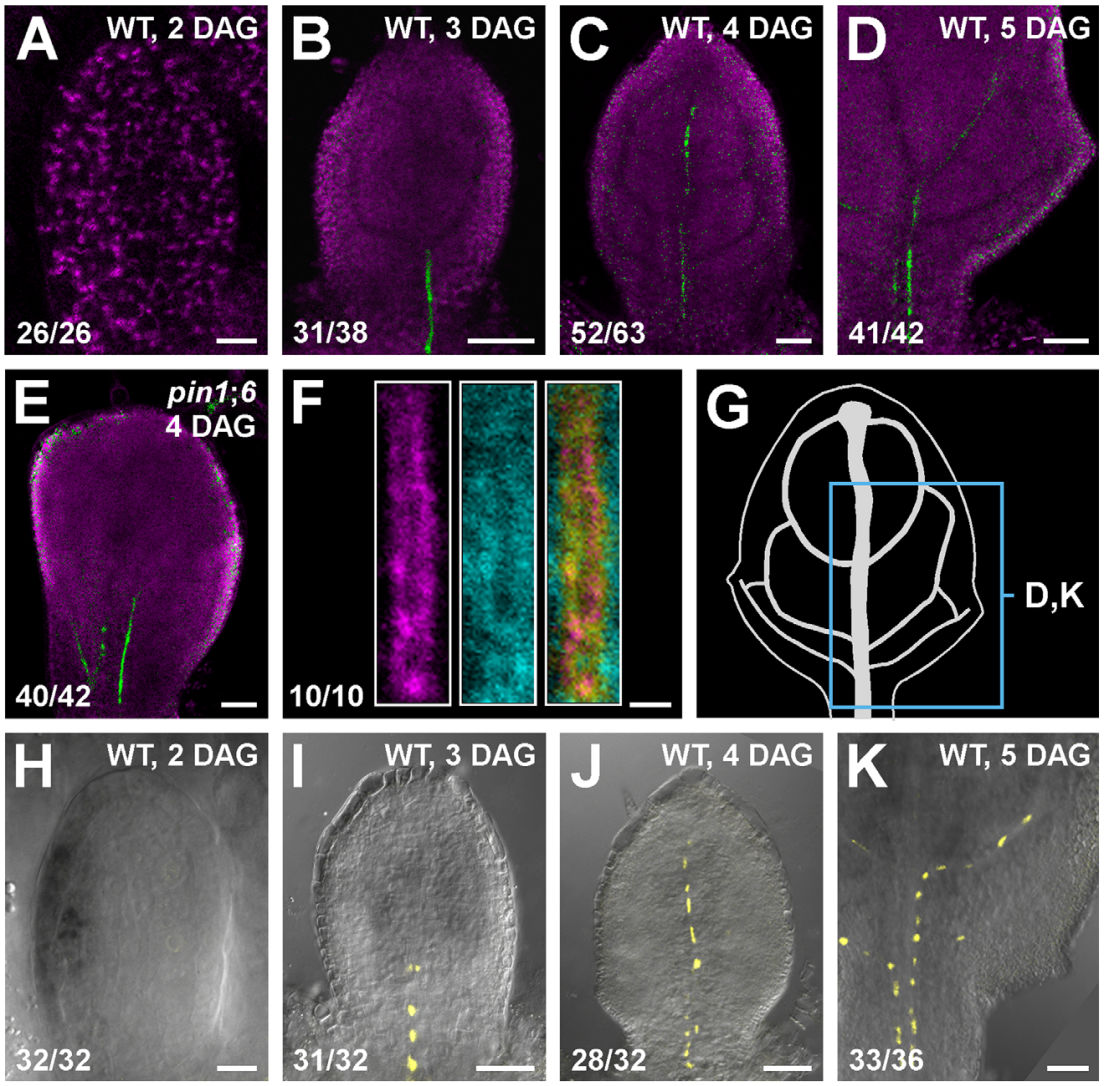
PIN8 expression in leaf development. (A–F,H–K) Top right: genotype, leaf age in days after germination (DAG). Bottom left: reproducibility index. Confocal laser scanning microscopy without (A–F) or with (H–K) transmitted light; first leaves. (A–E) Green: PIN8::PIN8:GFP expression; magenta: chlorophyll. (F) Expression at 3.25 DAG of PIN8::PIN8:GFP (left), staining by ER-Tracker Red (centre) and their overlay displayed with a dual-channel LUT (defined in Figure 2) (right). (G) 5-DAG first leaf illustrating positions of close-ups in (D) and (K). (H–K) PIN6::YFPnuc expression. Bars: (A,H) 10 µm; (B–E,I–K) 50 µm; (F) 2 µm.

Triple-mutant analysis suggests functions for *PIN8* in vein patterning (Figure 3; see Discussion). We thus asked whether *PIN8* was expressed, as *PIN1* [10], [11], *PIN5* [36] and *PIN6* (Figure 2 and Figure S2A–S2E), during vein development. To address this question, we imaged expression of a functional (Table S1) translational fusion of *PIN8* to GFP driven by the *PIN8* promoter (PIN8::PIN8:GFP). Expression of PIN8::PIN8:GFP was restricted to narrow sites of vein formation in both WT and *pin1*;*6* (Figure 4A–4E), suggesting PIN8 expression in vein development, a conclusion further supported by expression of a PIN8::YFPnuc transcriptional fusion (*PIN8* promoter driving expression of a nuclear YFP) (Figure 4H–4K).

In pollen, *PIN8* translational fusions localize to the ER [38], [39]. To determine PIN8 subcellular localization in leaves, we quantified degree of colocalization between expression of PIN8::PIN8:GFP and staining by the glibenclamide-derived ER-Tracker Red dye, which selectively stains the ER (e.g., [40]–[42]). Expression of PIN8::PIN8:GFP correlated with staining by ER-Tracker Red (Figure 4F) (mean Manders' coefficient ‘r’ ± SE: 0.82±0.03; *n* = 10) and overlapped with staining by the dapoxyl-derived ER-Tracker Blue-White DPX dye [43] (Figure S2G), suggesting ER-localization of PIN8 in vein development.

### Necessary functions of *PIN5*, *PIN6*, and *PIN8* in vein network formation

**Figure 5:**
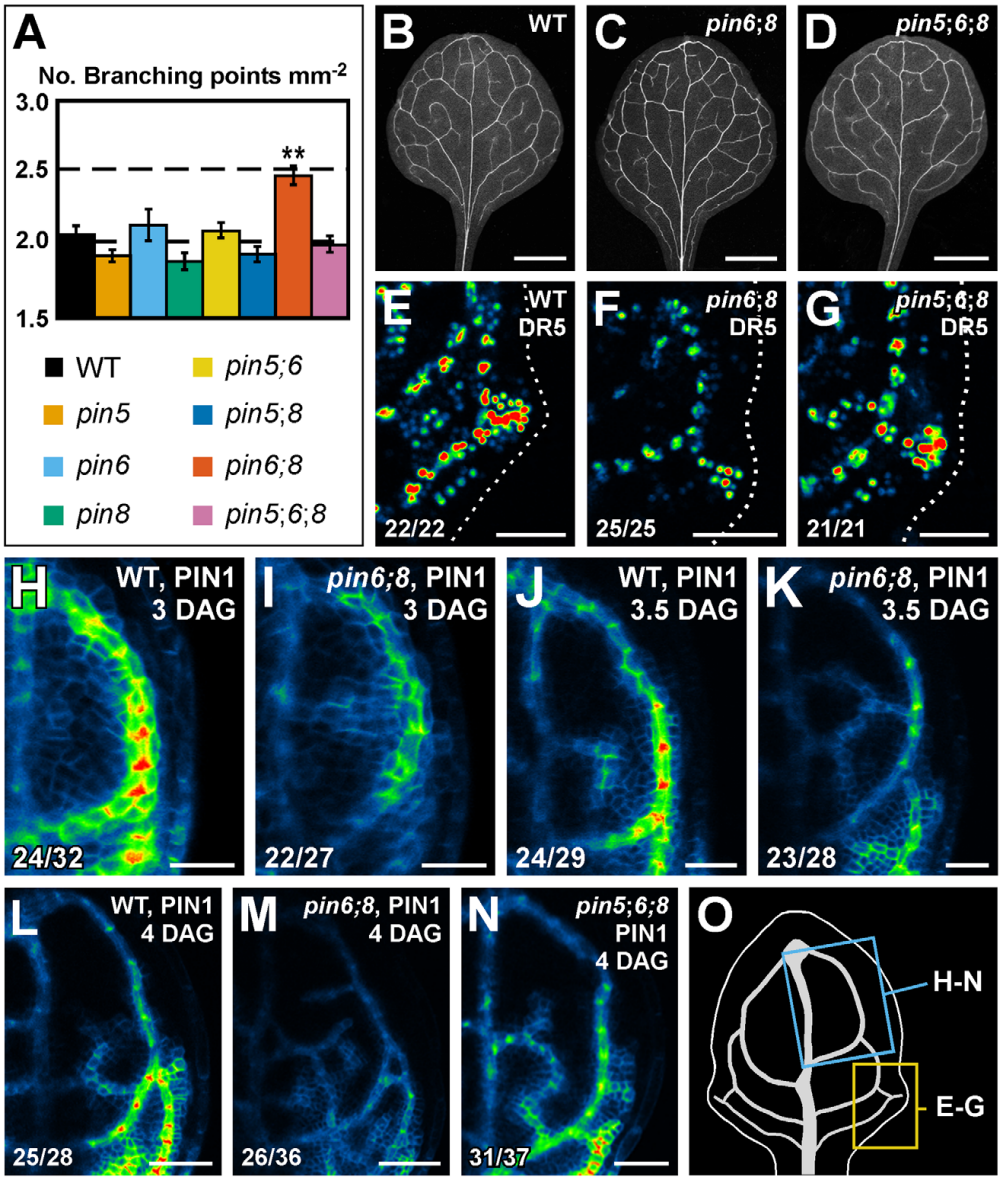
Necessity of *PIN5*, *PIN6*, and *PIN8* for vein network formation. (A) Vein network complexity as mean ± SE number of vein branching points per first-leaf area unit in mm^2^ [28]. Difference between *pin6*;*8* and all other genotypes was significant at *P*<0.01 (**) by one-way ANOVA and Tukey's test. Sample population sizes: WT, 30; *pin5*, 30; *pin6*, 30; *pin8*, 27; *pin5*;*6*, 28; *pin5*;*8*, 28; *pin6*;*8*, 28; *pin5*;*6*;*8*, 28. (B–N) Top right: genotype, markers and leaf age in days after germination (DAG). Bottom left: reproducibility index. (B–D) Dark-field illumination of cleared mature first leaves. (E–N) Confocal laser scanning microscopy; first leaves. LUT (in Figure 2K) visualizes expression levels. (E–G) DR5rev::YFPnuc expression, 4 DAG. Dotted line: leaf outline. (H–N) PIN1::PIN1:YFP expression. (O) 4-DAG first leaf illustrating positions of close-ups in (E–G) and (H–N). Bars: (B–D) 1.5 mm; (E–G,L–N) 50 µm; (H–K) 25 µm.

Single mutation of *PIN5*, *PIN6* or *PIN8* had no effect on vein patterns (Figure 1). Thus, to test whether a function in vein patterning could be assigned to ER-localized PIN proteins in the presence of PIN1-mediated intercellular auxin-transport, we analyzed vein patterns of combinations of *pin5*, *pin6* and *pin8*. Double and triple mutants had WT vein patterns (sample population sizes: WT, 26; *pin5*;*6*, 22; *pin5*;*8*, 24; *pin6*;*8*, 39; *pin5*;*6*;*8*, 30), but *pin6*;*8* had a more complex vein network, a defect that was normalized by *pin5* (Figure 5A–5D).

We next asked whether *pin6*;*8* defects in vein network formation were associated with changes in auxin response levels. To address this question, we imaged expression of the DR5rev::YFPnuc auxin response reporter [44]. In subepidermal tissues of WT leaves, DR5rev::YFPnuc was strongly expressed at sites of vein formation (Figure 5E); in subepidermal tissues of *pin6*;*8* leaves, DR5rev::YFPnuc expression was weaker (Figure 5F), a defect that was normalized by *pin5* (Figure 5G).

We then asked whether *pin6*;*8* defects in auxin response levels and vein network formation were associated with changes in auxin-responsive PIN1 expression [10], [44]–[46]. To address this question, we imaged expression of PIN1::PIN1:YFP [47] in the leaf area enclosed by the first loop. In both WT and *pin6*;*8*, PIN1::PIN1:YFP expression was initiated in broad domains that narrowed to sites of vein formation (Figure 5H–5M). However, in *pin6*;*8* PIN1::PIN1:YFP expression was weaker (Figure 5H–5M), vein-associated domains of PIN1::PIN1:YFP expression became visible at earlier time-points (Figure 5H and 5I), and at each time point we observed more vein-associated domains of PIN1::PIN1:YFP expression (Figure 5H–5M). Defects in PIN1::PIN1:YFP expression levels of *pin6*;*8* were normalized by *pin5* (Figure 5N).

In summary, *pin6*;*8* had defects in expression of DR5 and PIN1 and in formation of vein networks, and such defects were normalized by *pin5*.

### Sufficient functions of *PIN5*, *PIN6*, and *PIN8* in vein network formation

**Figure 6:**
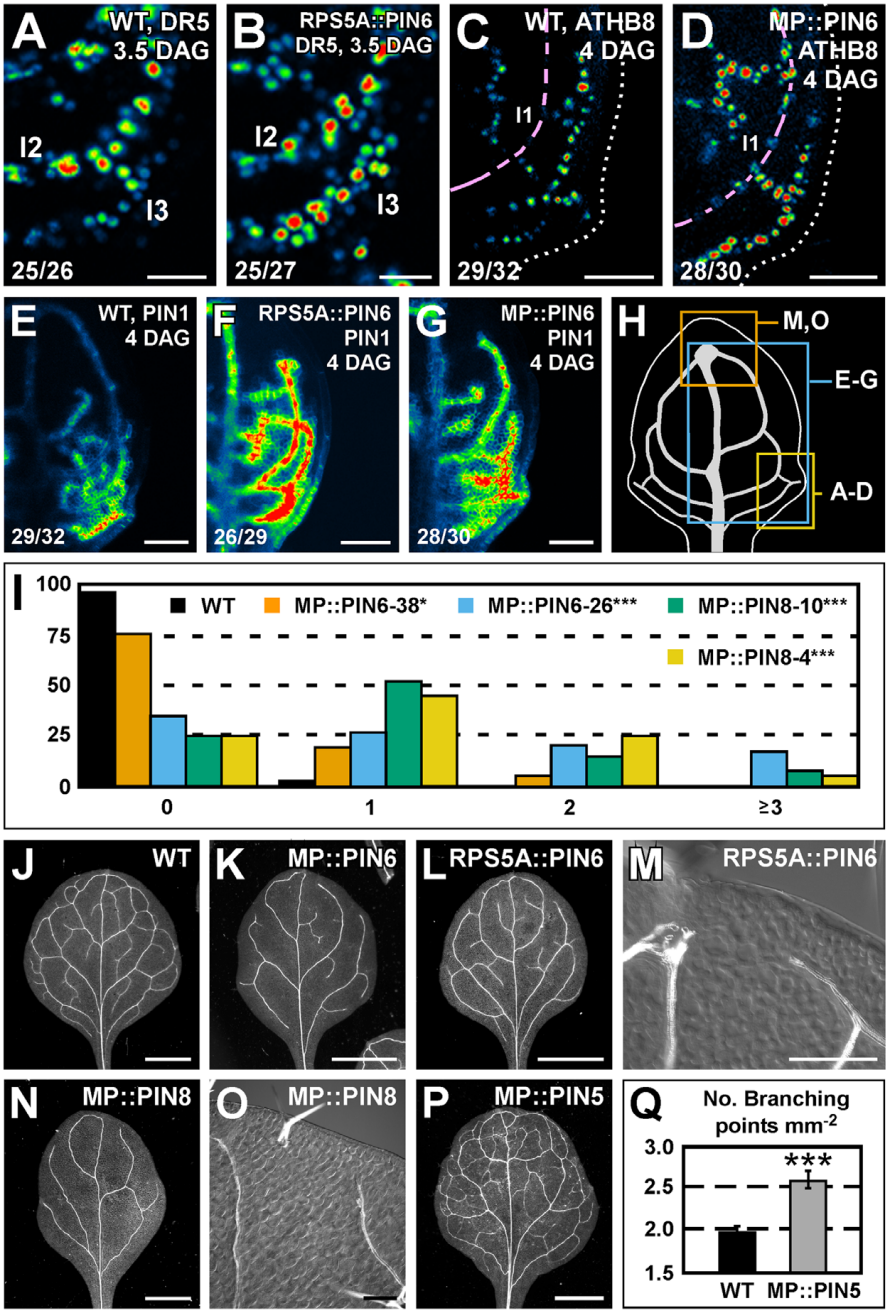
Sufficiency of *PIN5*, *PIN6*, and *PIN8* for vein network formation. (A–G,J–P) Top right: genotype, markers and leaf age in days after germination (DAG). Bottom left: reproducibility index. (A–G) Confocal laser scanning microscopy; first leaves. LUT (in Figure 2K) visualizes expression levels. (J–P) Dark-field (J–L,N,P) or differential-interference-contrast (M,O) illumination of cleared mature first leaves. (A,B) DR5rev::YFPnuc expression. (C,D) ATHB8::CFPnuc expression. Magenta line connects nuclei in the first loop (l1). Dotted line: leaf outline. (E–G) PIN1::PIN1:YFP expression. (H) 4-DAG first leaf illustrating positions of close-ups in (A–D), (E–G) and (M,O). (I) Percentage of first leaves with 0, 1, 2 or ≥3 open loops. Difference between MP::PIN6 and WT, and between MP::PIN8 and WT was significant at *P*<0.05 (*) or *P*<0.001 (***) by Kruskal-Wallis and Mann-Whitney test with Bonferroni correction. Sample population sizes: WT, 43; MP::PIN6-38, 40; MP::PIN6-26, 42; MP::PIN8-10, 40; MP::PIN8-4, 40. (Q) Vein network complexity as mean ± SE number of vein branching points per first-leaf area unit in mm^2^ [28]. Difference between MP::PIN5 and WT was significant at *P*<0.001 (***) by unpaired, two-tailed *t*-test. Sample population sizes: WT, 28; MP::PIN5, 28. l1, first loop; l2, second loop; l3, third loop. Bars: (A,B) 25 µm; (C–G) 50 µm; (J–L,N,P) 1.5 mm; (M,O) 0.2 mm.

We next asked whether *PIN6* function was sufficient to control expression of DR5 and PIN1 and formation of vein networks. To address this question, we expressed the *PIN6* cDNA by the promoter of *RIBOSOMAL PROTEIN S5A* (AT3G11940) (RPS5A::PIN6) and by the promoter of *MONOPTEROS* (AT1G19850) (MP::PIN6), each highly active in developing leaves [48] (Figure S2F). Expression of the auxin-responsive markers DR5rev::YFPnuc, ATHB8::CFPnuc [49] and PIN1::PIN1:YFP was stronger in leaves of RPS5A::PIN6 and MP::PIN6 (Figure 6A–6G). Furthermore, in both these backgrounds lateral domains of PIN1::PIN1:YFP expression often failed to join distal veins (Figure 6E–6G), defects that correlated with the simpler, open vein networks of the mature leaves (Figure 6I–6M).

We then asked whether ectopic expression of *PIN8* or *PIN5* had any effect on vein network formation. To address this question, we analyzed vein patterns of plants expressing the cDNA of *PIN8* or *PIN5* by the *MP* promoter (MP::PIN8 or MP::PIN5, respectively). We found that MP::PIN8 leaves had simpler, open vein networks and that MP::PIN5 leaves had a more complex vein network (Figure 6I and 6N–6Q).

In summary, ectopic *PIN6* expression was sufficient to control expression of DR5 and PIN1. Furthermore, ectopic expression of *PIN6* or *PIN8*, on the one hand, and of *PIN5*, on the other, had opposite effects on vein network formation.

### Simulation of *pin1*;*6* defects by reduction of auxin levels in *pin1*

**Figure 7:**
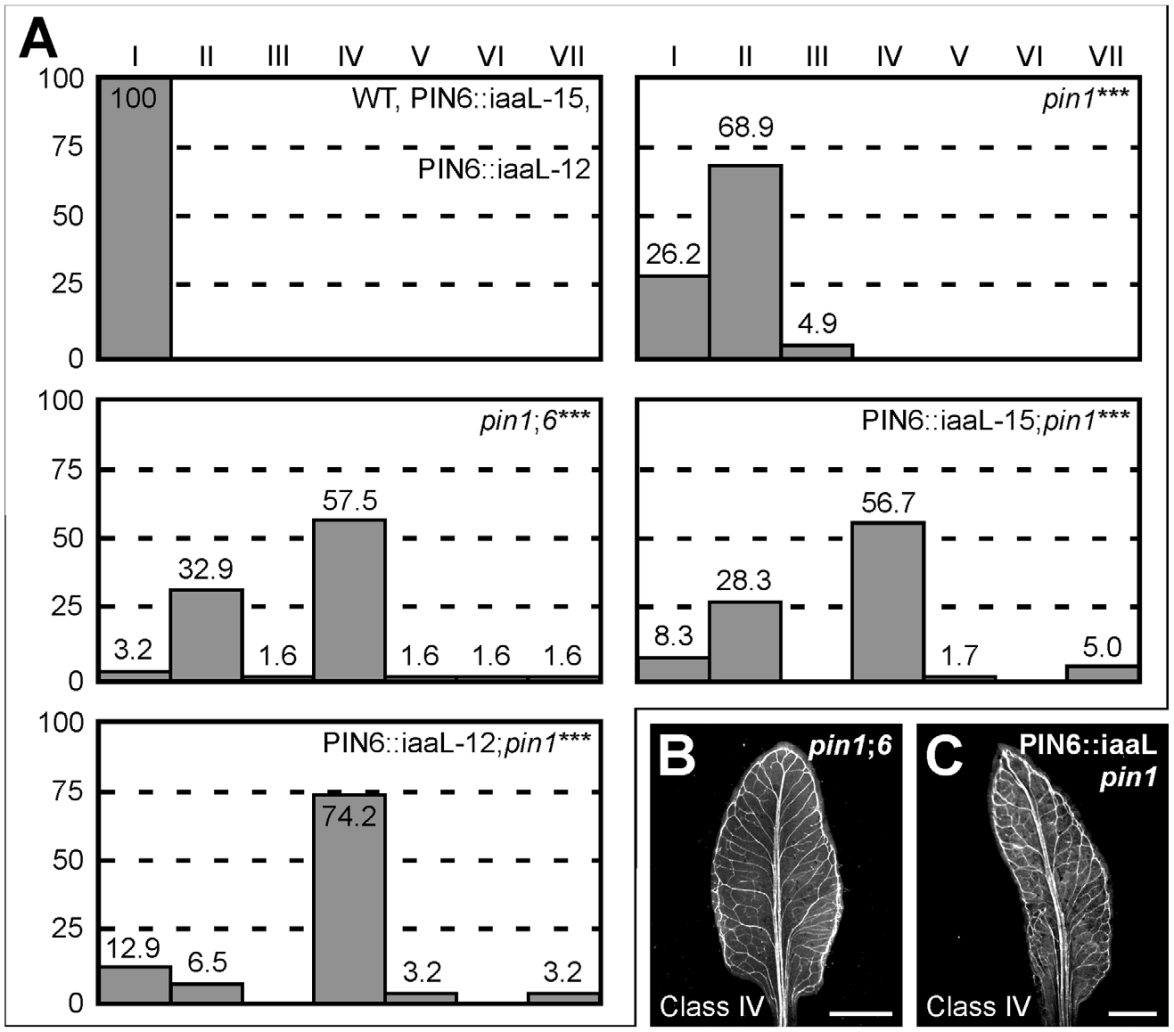
Control of *PIN1*-dependent vein patterning by intracellular auxin levels. (A) Percentages of leaves in phenotype classes (defined in Figure 1). Difference between *pin1* and WT, between *pin1*;*6* and *pin1*, and between PIN6::iaaL;*pin1* and *pin1* was significant at *P*<0.001 (***) by Kruskal-Wallis and Mann-Whitney test with Bonferroni correction. Sample population sizes: WT, 50; PIN6::iaaL-15, 50; PIN6::iaaL-12, 50; *pin1*, 61; *pin1*;*6*, 61; PIN6::iaaL-15;*pin1*, 60; PIN6::iaaL-12;*pin1*, 62. (B,C) Dark-field illumination of cleared mature first leaves. Top right: genotype. Bottom left: phenotype class. Bars: (B,C) 2 mm.

Weaker expression of auxin responsive markers in *pin6*;*8* leaves and stronger expression of auxin responsive markers in leaves of RPS5A::PIN6 and MP::PIN6 suggest that PIN6-mediated auxin transport increases intracellular levels of biologically active auxin (see Discussion). If this were the case, *pin1*;*6* phenotype spectrum might be mimicked by expressing in *pin1* the bacterial gene *iaaL*, which decreases levels of free auxin by its conjugation to lysine [50], [51]. Expression of PIN6::iaaL (*PIN6* promoter driving expression of iaaL) in *pin1* mimicked the phenotype spectrum of *pin1*;*6* leaves (Figure 7) and cotyledons (Figure S1), a finding that is consistent with the hypothesis that PIN6-mediated intracellular auxin transport increases auxin levels.

## Discussion

The mechanisms that control the patterning of vein networks of plant leaves have long fascinated biologists and mathematicians. Varied evidence has increasingly been accumulating that supports an inductive and orienting role for the transport of the plant signal auxin in leaf vein patterning [3, 5–7, 10–13], but molecular details have remained unclear. Here we show that the vein network of the Arabidopsis leaf is patterned by two distinct and convergent auxin-transport pathways: an intercellular pathway mediated at the plasma membrane (PM) by the PIN1 auxin transporter and an intracellular pathway mediated at the endoplasmic reticulum (ER) by the PIN6, PIN8 and PIN5 auxin transporters. While a role for other families of auxin transporters (e.g., [30]–[32]) in vein patterning is by no means precluded, our results suggest a new, unsuspected level of control of vein patterning by PIN-mediated auxin transport, one with repercussions on patterning of other plant features.

**Figure 8:**
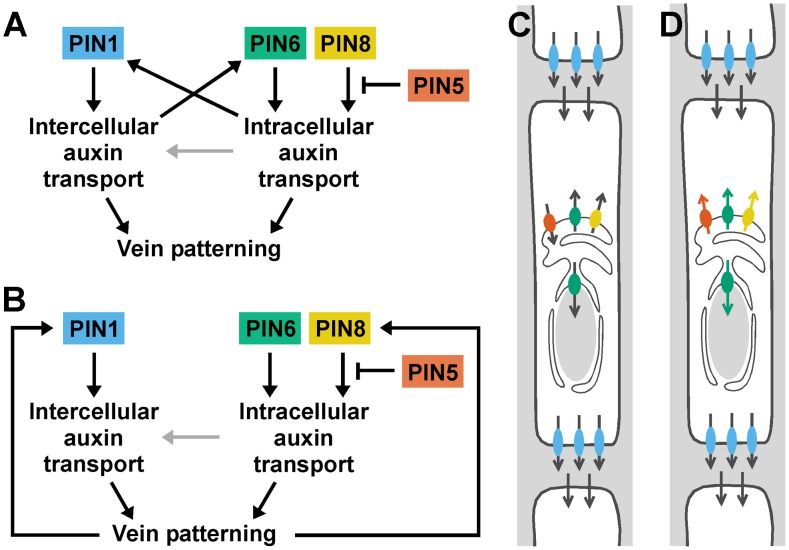
Summary and interpretation. (A,B) Two potential, non-mutually-exclusive regulatory circuits for *PIN1* and *PIN5*/*PIN6*/*PIN8* in vein patterning. Arrows indicate positive regulation; blunt-ended lines indicate negative regulation. Distinct functions of PIN1-mediated intercellular auxin transport and of PIN5/PIN6/PIN8-mediated intracellular auxin transport converge on vein patterning. Additionally, PIN1-mediated intercellular auxin transport could control vein patterning through regulation of PIN6 expression, and PIN5/PIN6/PIN8-mediated intracellular auxin transport could control vein patterning through regulation of PIN1 expression (A). Alternatively, vein patterning could feed back on expression of PIN1 and PIN6 (B). PIN1-independent functions of PIN5/PIN6/PIN8-mediated intracellular auxin transport in vein patterning could include intercellular auxin transport by a mechanism nonhomologous to that used by PM-localized PIN proteins [63] (grey arrows). (C,D) Localization of PIN1 (blue) at the basal plasma membrane and of PIN5 (orange), PIN6 (green) and PIN8 (yellow) at the endoplasmic reticulum/nuclear envelope (ER/NE) of vascular cells. For simplicity, PIN5, PIN6 and PIN8 are shown to be expressed in the same cell, only PIN6 is shown to be also localized at the NE, and localization of AUX/LAX [30], ABCB/PGP/MDR [32] and PILS [31] auxin transporters is not shown. Arrows indicate presumed directions of auxin transport. Antagonism between *pin5* and *pin6*;*8* in vein patterning could reflect opposite directions in which PIN5 and PIN6/PIN8 transport auxin (C), different abilities of PIN5 and PIN6/PIN8 to transport different auxins or auxin conjugates (orange, green and yellow arrows) (D; for simplicity, the mirror-image scenario, i.e. transport to the ER/NE lumen, is not shown), or varied combinations of the two. See text for additional details.

The localization of PIN1 to the PM [8] and of PIN6 to the ER together with the appearance in *pin1*;*6* of vein pattern defects that exceed the sum of the single-mutant defects suggest that *PIN1* and *PIN6* act in distinct auxin-transport pathways whose functions converge on vein patterning. By contrast, the overlapping subcellular localizations of PIN6 and PIN8 [36]–[39] together with the purely quantitative enhancement of *pin1*;*6* vein pattern defects by *pin8* suggest redundant function of *PIN6* and *PIN8* in *PIN1*-dependent vein patterning. Further, the overlapping subcellular localization of PIN8 [36]–[39] and PIN5 [36], [37], [39] together with the partial normalization of *pin1*;*6*;*8* vein pattern defects by *pin5* suggest antagonistic functions of *PIN8* and *PIN5* in *PIN1*/*PIN6*-dependent vein patterning. The interaction between the intercellular auxin-transport pathway controlled by PIN1 and the intracellular auxin-transport pathway controlled by PIN6, PIN8 and PIN5 is relevant for at least two other patterning events, separation of cotyledons and of leaves, and might have implications for other developmental processes. The redundancy between *PIN6* and *PIN8* and the antagonism between *PIN8* and *PIN5* are, however, independent of PIN1-mediated intercellular auxin transport: in the presence of *PIN1* function, *PIN6* and *PIN8* redundantly control vein network formation, and such redundant functions are antagonized by *PIN5*. Redundant functions of *PIN6* and *PIN8* and antagonistic functions of *PIN5*, as inferred from loss-of-function data, are also consistent with gain-of-function evidence: ectopic *PIN8* expression induces vein pattern defects similar to those induced by ectopic *PIN6* expression and opposite to those of *pin6*;*8*, suggesting that *PIN8* can supply vein patterning functions similar to those of *PIN6*; and ectopic *PIN5* expression induces vein pattern defects opposite to those induced by ectopic expression of *PIN6* or *PIN8* and similar to those of *pin6*;*8*, suggesting that *PIN5* can supply vein patterning functions opposite to those of *PIN6* and *PIN8*. The internally consistent relationship between the effects of loss of function of *PIN6*, *PIN8* and *PIN5* and the effects of gain of function of these genes is not limited to vein patterning but extends to auxin response: auxin response levels are decreased by simultaneous mutation of *PIN6* and *PIN8* and by ectopic expression of *PIN5* [36] and increased by ectopic expression of *PIN6* or *PIN8* [39] and by mutation of *PIN5* [36]. Thus, the genetic interaction between *PIN6*, *PIN8* and *PIN5* might reflect general properties of the mechanism with which these proteins function, a conclusion that is also supported by the antagonistic functions of *PIN8* and *PIN5* in a process unrelated to vein patterning such as pollen development [39]. The most parsimonious explanation for the antagonistic interaction between PIN6/PIN8 and PIN5, which transports auxin from the cytoplasm to the ER lumen [36], is that PIN6 and PIN8 transport auxin from the ER lumen to the cytoplasm or to the nucleus, whose envelope is continuous with the ER membrane [52], [53] (Figure 8C). This scenario is also supported by the observation that reducing auxin levels in *pin1* leads to *pin1*;*6* characteristic defects, and is consistent with higher levels of auxin measured in *PIN8* overexpressors [38], [39] and *pin5* mutants [36]. Alternatively, or in addition, PIN6, PIN8 and PIN5 could all transport in the same direction but have different affinities for auxins or auxin conjugates with different, even opposing, developmental functions (reviewed in [54]) (Figure 8D).

Our data suggest that auxin transported by PIN6 and PIN8 increases levels of auxin-responsive PIN1 expression during vein formation (Figure 8A). In *pin6*;*8*, low auxin levels at sites of vein formation could accelerate formation of narrow vein-associated domains of PIN1 expression, thus resulting in high-complexity vein networks [13], [55]–[58]. Conversely, in leaves ectopically expressing *PIN6*, high auxin levels at sites of vein formation could hinder or prevent formation and connection of vein-associated PIN1-expression domains, thus resulting in simple, open vein networks [13], [56], [57], [59]–[61]. Alternatively, PIN6/PIN8-mediated auxin transport could control vein network formation exclusively through PIN1-independent pathways; defects in auxin response or PIN1 expression would then be consequence, rather than cause, of defective vein-network formation (Figure 8B). In any case, the synthetic enhancement of *pin1* defects by *pin6* and *pin6*;*8*, as opposed to the epistasis of *pin6*;*8* to *pin1*, suggests vein patterning functions of PIN6/PIN8-mediated intracellular auxin transport beyond regulation of PIN1 expression or of intercellular auxin transport mediated by PM-localized PIN proteins (Figure 8A and 8B). We can probably exclude that such functions of PIN6 and PIN8 require physical interaction with PIN1 because PIN1 and PIN6/PIN8 localize to different cellular compartments and because *PIN1*/*pin1*;*pin6*/*pin6* lacks defects in cotyledons and vein patterns (*n*>50), defects that would be expected for interacting proteins (reviewed in [62]). Because the localization of PIN6 and PIN8 is maintained in *pin1* backgrounds, it seems unlikely that these two proteins have auxin transport functions that are redundant and homologous with those of PIN1. Nevertheless, PIN6/PIN8/PIN5-mediated intracellular auxin-transport could contribute to intercellular auxin transport by a mechanism nonhomologous to that used by PM-localized PIN proteins [63] (Figure 8A and 8B), a function of ER-localized PIN proteins that would be particularly relevant in primitive land plants like mosses, which seem to lack PM-localized PIN proteins [36] but do select cell files for vascular function [64].

## Materials and Methods

### Plants

Origin and nature of lines, genotyping strategies and oligonucleotide sequences are in Tables S1, S2, S3, respectively. Seeds were sterilized and germinated, and plants were grown and transformed as in [65].

### Imaging

For plasma-membrane stainings, seedlings were incubated in 10 µg ml^−1^ FM 4-64 (Invitrogen) for ∼4 min under vacuum before mounting. For endoplasmic-reticulum stainings, dissected leaves were incubated in 10 µM ER-Tracker Red (Invitrogen/Life Technologies) or ER-Tracker Blue-White DPX (Invitrogen/Life Technologies) for ∼30 min under vacuum before mounting. Leaf primordia were mounted in water under 0.17-mm-thick coverslips (Fischer Scientific) and imaged with the 20×/0.8 Plan-Apochromat or 40×/1.2 W C-Apochromat objective of an Axio Imager.M1/LSM 510 META confocal microscope (Carl Zeiss). 512×512-pixel frames were scanned unidirectionally at 8-bit depth with 1.6-µsec pixel dwell time and 8-fold averaging. Scanning zoom was adjusted to set pixel size to half the spacing of the features to be resolved, with minimum pixel size equal to half the objective lateral resolving power. Emission was collected from ∼5-µm-thick (single-fluorophore imaging) or ∼1-µm-thick (multi-fluorophore imaging) optical slices. Amplifier gain was set at 1; detector gain at ∼50–60%. Laser transmission and offset value were adjusted to match signal to detector's input range. Marker-line-specific imaging parameters are in Tables S4 and S5. For multi-fluorophore imaging, sequential excitation and collection of emission were performed in line-by-line channel-switching mode, which did not result in signal crossover under our imaging conditions. Signal levels in images acquired at identical settings were visualized as in [65]. Signal colocalization was visualized as in [66] and quantified as in [67]. Mature leaves were imaged as in [49]. Images were analyzed and processed with ImageJ (National Institutes of Health), and figures were generated with Canvas (ACD Systems International Inc.).

## Supporting Information

Figure S1 Cotyledon patterns of *pin* mutants. (A–G) Dark-field illumination of 4-day-old seedlings illustrating phenotype classes: two separate cotyledons (A); fused cotyledons and separate single cotyledon (B); three separate cotyledons (C); fused cotyledons (D); single cotyledon (E); partially fused cup-shaped cotyledon, side view; inset: top view (F); completely fused cup-shaped cotyledon, side view; inset: top view (G). (H) Percentages of seedlings in phenotype classes. Difference between *pin1* and WT, between *pin1*;*6* and *pin1*, between UBQ10::amiPIN6-10;*pin1* and *pin1*, between *pin1*;*6*;*8* and *pin1*;*6*, and between *pin1;5;6;8* and *pin1;6;8*, and between PIN6::iaaL;*pin1* and *pin1* was significant at *P*<0.001 (***) by Kruskal-Wallis and Mann-Whitney test with Bonferroni correction. Sample population sizes: WT, 50; *pin2*, 50; *pin3*, 50; *pin4*, 50; *pin5*, 50; *pin6*, 50; UBQ10::amiPIN6-5, 50; UBQ10::amiPIN6-10, 50; *pin7*, 50; *pin8*, 50; PIN6::iaaL-15, 50; PIN6::iaaL-12, 50; *pin1*, 61; *pin1*;*2*, 53 *pin1*;*3*, 45; *pin1*;*4*, 49; *pin1*;*5*, 47; *pin1*;*6*, 56; UBQ10::amiPIN6-5;*pin1*, 50; UBQ10::amiPIN6-10;*pin1*, 74; *pin1*;*7*, 49; *pin1*;*8*, 62; *pin1*;*5*;*6*, 54; *pin1*;*6*;*8*, 52; *pin1*;*5*;*6*;*8*, 50; PIN6::iaaL-15;*pin1*, 53; PIN6::iaaL-12;*pin1*, 51. Bars: (A–C,E) 1 mm; (D,F) 0.4 mm; (G) 0.25 mm.
(TIF)

Figure S2 Expression of PIN6, PIN8 and *MP* in leaf development. (A–G) Confocal laser scanning microscopy with (A–C,E,F) or without (D,G) transmitted light. Top right: reporters and leaf age in days after germination (DAG). Bottom left: reproducibility index. First leaves. (D,E) Blue: chlorophyll. (E) Close-up of area as boxed in D. (G) Expression at 3.25 DAG of PIN8::PIN8:GFP (left), staining by ER-Tracker Blue-White DPX (centre) and their overlay displayed with a dual-channel LUT (defined in Figure 2) (right). e, epidermis. Bars: (A,B) 10 µm; (C,F) 50 µm; (D) 100 µm; (E) 20 µm; (G) 2 µm.
(TIF)

Figure S3 Colocalization analysis of PIN6 with endoplasmic-reticulum or plasma-membrane markers. (A–O) Top right: marker. Bottom left: reproducibility index. Confocal laser scanning microscopy. First leaves. Expression of J1721::GFPer (A,M), 35S::YFPer (B,E), ATHB8::GFPnuc (D), PIN6::PIN6:GFP (G), 35S::RTNLB4:YFP (H), 35S::YFPpm (J,N), FM4-64 (K) and respective overlays displayed with a dual-channel LUT (defined in Figure 2) (C,F,I,L,O). Bars: (A–L) 5 µm; (M–O) 2.5 µm.
(TIF)

Table S1 Origin and nature of lines.
(DOC)

Table S2 Genotyping strategies.
(DOC)

Table S3 Oligonucleotide sequences.
(DOC)

Table S4 Imaging parameters: single-marker lines.
(DOC)

Table S5 Imaging parameters: multi-marker lines.
(DOC)
