# Supplementary figures and images for: Patterning of Leaf Vein Networks by Convergent Auxin Transport Pathways
Source: PLoS Genet. 2013 Feb 21;9(2):e1003294. doi: 10.1371/journal.pgen.1003294 (PMC3578778; doi:10.1371/journal.pgen.1003294)

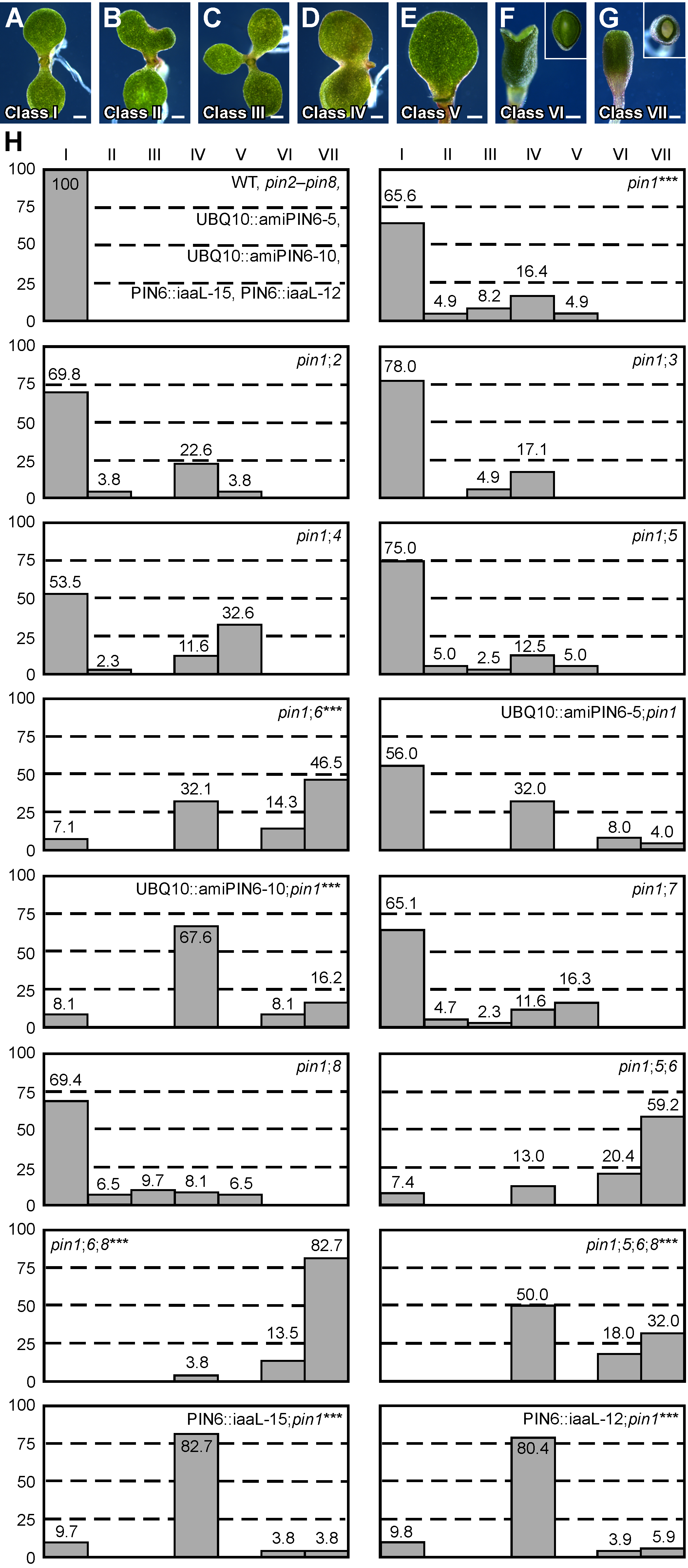

Supplement: Figure S1 — Cotyledon patterns of pin mutants. (A–G) Dark-field illumination of 4-day-old seedlings illustrating phenotype classes: two separate cotyledons (A); fused cotyledons and separate single cotyledon (B); three separate cotyledons (C); fused cotyledons (D); single cotyledon (E); partially fused cup-shaped cotyledon, side view; inset: top view (F); completely fused cup-shaped cotyledon, side view; inset: top view (G). (H) Percentages of seedlings in phenotype classes. Difference between pin1 and WT, between pin1;6 and pin1, between UBQ10::amiPIN6-10;pin1 and pin1, between pin1;6;8 and pin1;6, and between pin1;5;6;8 and pin1;6;8, and between PIN6::iaaL;pin1 and pin1 was significant at P<0.001 (***) by Kruskal-Wallis and Mann-Whitney test with Bonferroni correction. Sample population sizes: WT, 50; pin2, 50; pin3, 50; pin4, 50; pin5, 50; pin6, 50; UBQ10::amiPIN6-5, 50; UBQ10::amiPIN6-10, 50; pin7, 50; pin8, 50; PIN6::iaaL-15, 50; PIN6::iaaL-12, 50; pin1, 61; pin1;2, 53 pin1;3, 45; pin1;4, 49; pin1;5, 47; pin1;6, 56; UBQ10::amiPIN6-5;pin1, 50; UBQ10::amiPIN6-10;pin1, 74; pin1;7, 49; pin1;8, 62; pin1;5;6, 54; pin1;6;8, 52; pin1;5;6;8, 50; PIN6::iaaL-15;pin1, 53; PIN6::iaaL-12;pin1, 51. Bars: (A–C,E) 1 mm; (D,F) 0.4 mm; (G) 0.25 mm. (TIF) [file pgen.1003294.s001.tif]

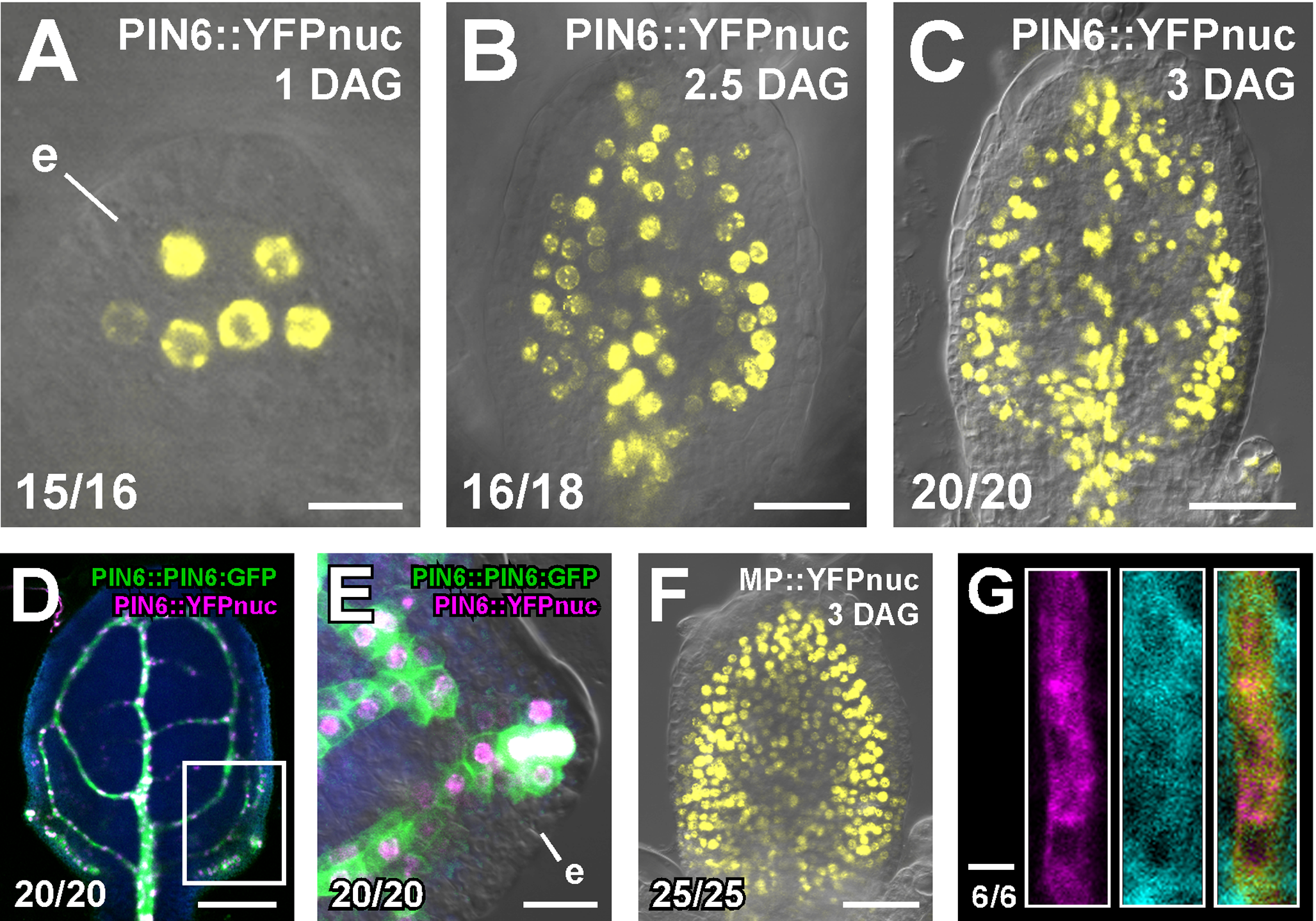

Supplement: Figure S2 — Expression of PIN6, PIN8 and MP in leaf development. (A–G) Confocal laser scanning microscopy with (A–C,E,F) or without (D,G) transmitted light. Top right: reporters and leaf age in days after germination (DAG). Bottom left: reproducibility index. First leaves. (D,E) Blue: chlorophyll. (E) Close-up of area as boxed in D. (G) Expression at 3.25 DAG of PIN8::PIN8:GFP (left), staining by ER-Tracker Blue-White DPX (centre) and their overlay displayed with a dual-channel LUT (defined in Figure 2) (right). e, epidermis. Bars: (A,B) 10 µm; (C,F) 50 µm; (D) 100 µm; (E) 20 µm; (G) 2 µm. (TIF) [file pgen.1003294.s002.tif]

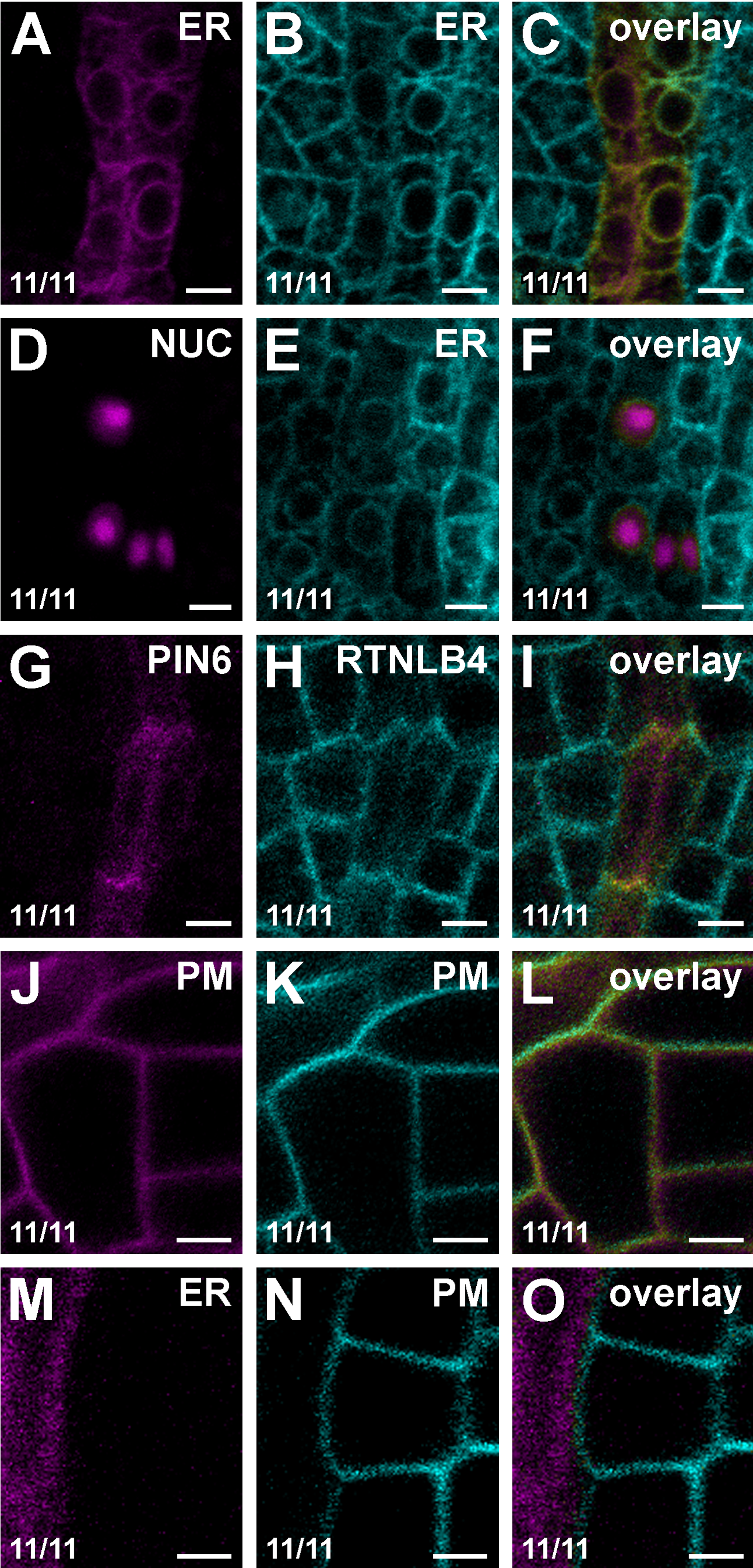

Supplement: Figure S3 — Colocalization analysis of PIN6 with endoplasmic-reticulum or plasma-membrane markers. (A–O) Top right: marker. Bottom left: reproducibility index. Confocal laser scanning microscopy. First leaves. Expression of J1721::GFPer (A,M), 35S::YFPer (B,E), ATHB8::GFPnuc (D), PIN6::PIN6:GFP (G), 35S::RTNLB4:YFP (H), 35S::YFPpm (J,N), FM4-64 (K) and respective overlays displayed with a dual-channel LUT (defined in Figure 2) (C,F,I,L,O). Bars: (A–L) 5 µm; (M–O) 2.5 µm. (TIF) [file pgen.1003294.s003.tif]
