# Supplementary material for: Patterning of Leaf Vein Networks by Convergent Auxin Transport Pathways
Source: PLoS Genet. 2013 Feb 21;9(2):e1003294. doi: 10.1371/journal.pgen.1003294 (PMC3578778; doi:10.1371/journal.pgen.1003294)
Supplement: Table S1 — Origin and nature of lines. (DOC) [file pgen.1003294.s004.doc]

**Table S1.** Origin and nature of lines.

| **Line** | **Origin/Nature** |
| --- | --- |
| *pin1-1* | [1,2]; WT at the *TTG1* (AT5G24520) locus |
| *eir1-1 (pin2)* | [3,4] |
| *pin3-3* | [5] |
| *pin4-2* | [6] |
| *pin5-4* | [7] |
| *pin6* | SM_3_15050 (ABRC) [8] containing a single dSpm transposable element at position +183 of *PIN6* (AT1G77110) |
| UBQ10::amiPIN6 | Transcriptional fusion of *UBQ10* (AT4G05320; -1516 to –1; primers: ‘UBQ10 HindIII Forw’ and ‘UBQ10 SmaI Rev’) to an artificial microRNA [9] targeting *PIN6* (AT1G77110; primers: ‘PIN6 I miR-s’, ‘PIN6 II miR-a’, ‘PIN6 III miR*s’, ‘PIN6 IV miR*a’, ‘pRS300 A’ and ‘pRS300 B’) |
| *pin7^En^* | [10] |
| *pin8-1* | [11] |
| PIN6::PIN6:GFP | Translational fusion of *PIN6* (AT1G77110; -3784 to +4252; primers: 'PIN6 prom SmaI forw' and 'PIN6 2179 SphI rev', 'PIN6 2180 EcoRI forw' and 'PIN6 UTR XhoI rev') to EGFP (Clontech; insertion at +2179 of *PIN6*; primers: 'EGFP PstI SphI forw' and 'EGFP EcoRI rev'); reverts the cotyledon phenotype of *pin1*;*6* to that of *pin1* |
| PIN6::YFPnuc | Transcriptional fusion of *PIN6* (AT1G77110; -3747 to -6; primers: 'PIN6 transc forw' and 'PIN6 transc rev') to HTA6:EYFP [12] |
| PIN1::PIN1:CFP | [13] |
| 35S::YFPer | [14] |
| 35S::RTNLB4:YFP | [15] |
| J1721::GFPer | [16,17] |
| ATHB8::GFPnuc | Transcriptional fusion of *ATHB8* (AT4G32880; -1997 to -1; primers: 'ATHB8 attB1F' and 'ATHB8 attB2R') to EGFP:nuc [18] |
| 35S::YFPpm | [19] |
| PIN8::PIN8:GFP | Translation fusion of *PIN8* (AT5G15100; -1812 to +2443; primers: ‘PIN8 prom BamHI forw’ and ‘PIN8 UTR SacI Rev’) to EGFP (Clontech; insertion at +684 of *PIN8*; primers: 'EGFP BglII forw' and 'EGFP BglII rev'); reverts the cotyledon phenotype of *pin1*;*6*;*8* to that of *pin1*;*6* |
| PIN8::YFPnuc | Transcriptional fusion of *PIN8* (AT5G15100; -1812 to -1; primers: ‘PIN8 transc forw’ and ‘PIN8 transc rev’) to HTA6:EYFP [12] |
| DR5rev::YFPnuc | [20]; transformed into Col-0 |
| PIN1::PIN1:YFP | [21] |
| MP::PIN6 | Transcriptional fusion of *MP* (AT1G18950; -3282 to -1; primers: ‘MP SalI Fwd’ and ‘MP BamHI Rev’) to *PIN6* coding sequence (AT1G77110; primers: 'PIN6OX SmaI forw' and 'PIN6OX Ecl136II rev') |
| MP::YFPnuc | Transcriptional fusion of *MP* (AT1G18950; -3281 to –1; primers: ‘MP prom Gateway Fwd’ and ‘MP prom Gateway Rev’) to HTA6:EYFP [12] |
| ATHB8::CFPnuc | [17] |
| RPS5A::PIN6 | Transcriptional fusion of *RPS5A* (AT3G11940; -2236 to –1; primers: ‘RPS5A SmaI Forw’ and ‘RPS5A SmaI Rev’) to *PIN6* coding sequence (AT1G77110; primers: 'PIN6OX SmaI forw' and 'PIN6OX Ecl136II rev') |
| MP::PIN8 | Transcriptional fusion of *MP* (AT1G18950; -3282 to -1; primers: ‘MP SalI Fwd’ and ‘MP BamHI Rev’) to *PIN8* coding sequence (AT5G15100; primers: ‘PIN8OX KpnI forw’ and 'PIN8OX BamHI rev') |
| MP::PIN5 | Transcriptional fusion of *MP* (AT1G18950; -3282 to -1; primers: ‘MP SalI Fwd’ and ‘MP BamHI Rev’) to *PIN5* coding sequence (AT5G16530; primers: ‘PIN5OX SmaI forw’ and ‘PIN5OX BamHI rev 2’) |
| PIN6::iaaL | Transcriptional fusion of *PIN6* (AT1G77110; -3784 to -1; Primers: 'PIN6 prom SalI F' and 'PIN6 prom BamHI R') to iaaL coding sequence [22] (primers: 'IAAL BamHI F' and 'IAAL KpnI R') |

**Supplemental References**

1. Goto N, Starke M, Kranz AR. (1987) Effect of gibberellins on flower development of the pin-formed mutant of Arabidopsis thaliana. Arabidopsis Information Service 23: 66-71.

2. Gälweiler L, Guan C, Muller A, Wisman E, Mendgen K, et al. (1998) Regulation of polar auxin transport by AtPIN1 in Arabidopsis vascular tissue. Science 282: 2226-2230.

3. Roman G, Lubarsky B, Kieber JJ, Rothenberg M, Ecker JR. (1995) Genetic analysis of ethylene signal transduction in Arabidopsis thaliana: Five novel mutant loci integrated into a stress response pathway. Genetics 139: 1393-1409.

4. Luschnig C, Gaxiola RA, Grisafi P, Fink GR. (1998) EIR1, a root-specific protein involved in auxin transport, is required for gravitropism in Arabidopsis thaliana. Genes & Development 12: 2175-2187.

5. Friml J, Wisniewska J, Benkova E, Mendgen K, Palme K. (2002) Lateral relocation of auxin efflux regulator PIN3 mediates tropism in Arabidopsis. Nature 415: 806-809.

6. Friml J, Benková E, Blilou I, Wisniewska J, Hamann T, et al. (2002) AtPIN4 mediates sink-driven auxin gradients and root patterning in Arabidopsis. Cell 108: 661–673.

7. Mravec J, Skupa P, Bailly A, Hoyerova K, Krecek P, et al. (2009) Subcellular homeostasis of phytohormone auxin is mediated by the ER-localized PIN5 transporter. Nature 459: 1136-1140.

8.     Tissier AF, Marillonnet S, Klimyuk V, Patel K, Torres MA, et al. (1999) Multiple independent defective suppressor-mutator transposon insertions in Arabidopsis: A tool for functional genomics. Plant Cell 11: 1841-1852.

9. Schwab R, Ossowski S, Riester M, Warthmann N, Weigel D. (2006) Highly specific gene silencing by artificial microRNAs in Arabidopsis Plant Cell 18: 1121-1133.

10. Blilou I, Xu J, Wildwater M, Willemsen V, Paponov I, et al. (2005) The PIN auxin efflux facilitator network controls growth and patterning in Arabidopsis roots. Nature 433: 39-44.

11. Bosco CD, Dovzhenko A, Liu X, Woerner N, Rensch T, et al. (2012) The endoplasmic reticulum localized PIN8 is a pollen specific auxin carrier involved in intracellular auxin homeostasis. The Plant Journal 71: 860-870.

12. Zhang C, Gong FC, Lambert GM, Galbraith DW. (2005) Cell type-specific characterization of nuclear DNA contents within complex tissues and organs. Plant Methods 1: 7. doi:10.1186/1746-4811-1-7.

13. Gordon SP, Heisler MG, Reddy GV, Ohno C, Das P, et al. (2007) Pattern formation during de novo assembly of the Arabidopsis shoot meristem. Development 134: 3539-3548.

14. Nelson BK, Cai X, Nebenfuhr A. (2007) A multicolored set of in vivo organelle markers for co-localization studies in Arabidopsis and other plants. The Plant Journal 51: 1126-1136.

15. Nziengui H, Bouhidel K, Pillon D, Der C, Marty F, et al. (2007) Reticulon-like proteins in Arabidopsis thaliana: Structural organization and ER localization FEBS Letters 581: 3356-3362.

16. Haseloff J. (1999) GFP variants for multispectral imaging of living cells. Methods in Cell Biology 58: 139-151.

17. Sawchuk MG, Head P, Donner TJ, Scarpella E. (2007) Time-lapse imaging of Arabidopsis leaf development shows dynamic patterns of procambium formation. New Phytologist 176: 560-571.

18. Kubo M, Udagawa M, Nishikubo N, Horiguchi G, Yamaguchi M, et al. (2005) Transcription switches for protoxylem and metaxylem vessel formation. Genes & Development 19: 1855-1860.

19. Cutler SR, Ehrhardt DW, Griffitts JS, Somerville CR. (2000) Random GFP::CDNA fusions enable visualization of subcellular structures in cells of arabidopsis at a high frequency. Proceedings of the National Academy of Sciences, USA 97: 3718-3723.

20. Heisler MG, Ohno C, Das P, Sieber P, Reddy GV, et al. (2005) Patterns of auxin transport and gene expression during primordium development revealed by live imaging of the Arabidopsis inflorescence meristem. Current Biology 15: 1899-1911.

21. Xu J, Hofhuis H, Heidstra R, Sauer M, Friml J, et al. (2006) A molecular framework for plant regeneration. Science 311: 385-388.

22. Roberto FF, Klee H, White F, Nordeen R, Kosuge T. (1990) Expression and fine structure of the gene encoding N epsilon-(indole-3-acetyl)-L-lysine synthetase from Pseudomonas savastanoi. Proceedings of the National Academy of Sciences, USA 87: 5797-5801.
