# Supplementary material for: Patterning of Leaf Vein Networks by Convergent Auxin Transport Pathways
Source: PLoS Genet. 2013 Feb 21;9(2):e1003294. doi: 10.1371/journal.pgen.1003294 (PMC3578778; doi:10.1371/journal.pgen.1003294)
Supplement: Table S2 — Genotyping strategies. (DOC) [file pgen.1003294.s005.doc]

**Table S2.** Genotyping strategies.

| **Line** | **Strategy** |
| --- | --- |
| *pin1-1* | 'pin1-1 F' and 'pin1-1 R'; *Tat*I |
| *eir1-1* | 'eir1-1 F' and 'eir1-1 R'; *Bse*LI |
| *pin3-3* | 'pin3-3 F' and 'pin3-3 R'; *Sty*I |
| *pin4-2* | *PIN4*:'PIN4 forw geno II' and 'PIN4en rev Ikram'; *pin4*: 'PIN4en rev Ikram' and 'en primer' |
| *pin5-4* | *PIN5*: 'SALK_042994 LP' and 'SALK_042994 RP'; *pin5*: 'SALK_042994 RP' and 'LBb1.3' |
| *pin6* | *PIN6*: 'PIN6 spm F' and 'PIN6 spm R'; *pin6*: 'PIN6 spm F' and 'Spm32' |
| *pin7^En^* | *PIN7*:'PIN7en forw Ikram' and 'PIN7en rev'; *pin7*: 'PIN7en rev Ikram II' and 'en primer' |
| *pin8-1* | *PIN8*: 'SALK_107965 LP' and 'SALK_107965 RP'; *pin8*: 'SALK_107965 RP' and 'LBb1.3' |
