# Supplementary material for: Patterning of Leaf Vein Networks by Convergent Auxin Transport Pathways
Source: PLoS Genet. 2013 Feb 21;9(2):e1003294. doi: 10.1371/journal.pgen.1003294 (PMC3578778; doi:10.1371/journal.pgen.1003294)
Supplement: Table S3 — Oligonucleotide sequences. (DOC) [file pgen.1003294.s006.doc]

**Table S3.** Oligonucleotide sequences.

| **Name** | **Sequence (5' to 3')** |
| --- | --- |
| pin1-1 F | ATGATTACGGCGGCGGACTTCTA |
| pin1-1 R | TTCCGACCACCACCAGAAGCC |
| eir1-1 F | TTGTTGATCATTTTACCTGGGACA |
| eir1-1 R | GGTTGCAATGCCATAAATAGAC |
| pin3-3 F | GGAGCTCAAACGGGTCACCCG |
| pin3-3 R | GCTGGATGAGCTACAGCTATATTC |
| en primer | GAGCGTCGGTCCCCACACTTCTATAC |
| PIN4 forw geno II | GTCCGACTCCACGGCCTTC |
| PIN4en rev Ikram | ATCTTCTTCTTCACCTTCCACTCT |
| LBb1.3 | ATTTTGCCGATTTCGGAAC |
| SALK_042994 LP | TGTGGTTGTGGGAGAGAAGTC |
| SALK_042994 RP | AAATTTGGACTTACGCTGTGC |
| Spm32 | TACGAATAAGAGCGTCCATTTTAGAGTG |
| PIN6 spm F | CATAACGAAGCTAACTAAGGGGTAATCTC |
| PIN6 spm R | GGAGTTCAAAGAGGAATAGTAGCAGAG |
| UBQ10 HindIII Forw | CTCAAGCTTTCCCATGTTTCTCGTCTGTC |
| UBQ10 SmaI Rev | CGACCCGGGCTGTTAATCAGAAAAACTCAG |
| PIN6 I miR-s | GATTAAAGATACTATCATCCCACTCTCTCTTTTGTATTCC |
| PIN6 II miR-a | GAGTGGGATGATAGTATCTTTAATCAAAGAGAATCAATGA |
| PIN6 III miR*s | GAGTAGGATGATAGTTTCTTTATTCACAGGTCGTGATATG |
| PIN6 IV miR*a | GAATAAAGAAACTATCATCCTACTCTACATATATATTCCT |
| pRS300 A | CTGCAAGGCGATTAAGTTGGGTAAC |
| pRS300 B | GCGGATAACAATTTCACACAGGAAACAG |
| PIN7en forw Ikram | CCTAACGGTTTCCACACTCA |
| PIN7en rev | TAGCTCTTTAGGGTTTAGCTC |
| PIN7en rev Ikram II | GGTTTAGCTCTGCTGTGGAGTT |
| SALK_107965 LP | TGAAAGACATTTTGATGGCATC |
| SALK_107965 RP | CCAAATCAAGCTTTGCAAGAC |
| PIN6 prom SmaI forw | ATTCCCGGGGATGATTGTTTAAGATAAGTTGCAAAG |
| PIN6 2179 SphI rev | ATAGCATGCTTGCTTGCCAATCGCAGCCACC |
| PIN6 2180 EcoRI forw | ATGGAATTCGAAATGCCAAGTGCAATTGTAATGATG |
| PIN6 UTR XhoI rev | ATACTCGAGTCGAATGCTTTTGGTTTCGG |
| EGFP PstI SphI forw | TATCTGCAGGCATGCGCAGTGAGCAAGGGCGAGGAGCTG |
| EGFP EcoRI rev | TATGAATTCCTTGTACAGCTCGTCCATGCCG |
| PIN6 transc forw | GGGGACAAGTTTGTACAAAAAAGCAGGCTGCTCGGATTATATTTATATG |
| PIN6 transc rev | GGGGACCACTTTGTACAAGAAAGCTGGGTTCTTTGCCTCTTCTTCTTC |
| ATHB8 attB1F | GGGGACAAGTTTGTACAAAAAAGCAGGCTGACGATAATGATGATAACTAC |
| ATHB8 attB2R | GGGGACCACTTTGTACAAGAAAGCTGGGTCTTTGATCCTCTCCGATCTCTC |
| PIN8 prom BamHI forw | ATTGGATCCGAATTTTGTAAAATGTGAAGG |
| PIN8 UTR SacI Rev | ATAGAGCTCGGGAAGAAGAAAGGGTAAC |
| EGFP BglII forw | TATAGATCTGTGAGCAAGGGCGAGGAG |
| EGFP BglII rev | TATAGATCTCTTGTACAGCTCGTCCATGC |
| PIN8 transc forw | GGGGACAAGTTTGTACAAAAAAGCAGGCTGAATTTTGTAAAATGTGAAG |
| PIN8 transc rev | GGGGACCACTTTGTACAAGAAAGCTGGGTGTTTTTATCAAATTGTACAATAC |
| MP SalI Fwd | TATGTCGACCCCGGGTTAATCAGTATTATTAC |
| MP BamHI Rev | TAGGGATCCACAGAGAGATTTTTCAATGTTCTG |
| PIN6OX SmaI forw | ATACCCGGGATGATAACGGGAAACGAATTCTAC |
| PIN6OX Ecl136II rev | ATTGAGCTCTCATAGGCCCAAGAGGACG |
| MP prom Gateway Fwd | GGGGACAAGTTTGTACAAAAAAGCAGGCTCCGGGTTAATCAGTATTATTAC |
| MP prom Gateway Rev | GGGGACCACTTTGTACAAGAAAGCTGGGTACAGAGAGATTTTTCAATGTTCTG |
| RPS5A SmaI Forw | ATACCCGGAGCAGGAGATCTATCAGTG |
| RPS5a SmaI Rev | ATACCCGGGGGCTGTGGTGAGAGAAAC |
| PIN8OX KpnI forw | TATGGTACCATGATCTCCTGGCTCGATATCTAC |
| PIN8OX BamHI rev | TATGGATCCTCATAGGTCCAATAGAAAATAATATGC |
| PIN5OX SmaI forw | ATACCCGGGATGATAAATTGTGGAGATGTTTAC |
| PIN5OX BamHI rev 2 | AT GGATCCTCAATGAATAAACTCCAGAGC |
| PIN6 prom SalI F | GCGGTCGACTGATGATTGTTTAAGATAAG |
| PIN6 prom BamHI R | TCTGGATCCTCTTTGCCTCTTCTTCTTC |
| IAAL BamHI F | ATAGGATCCATGACTGCCTACGATATGGAAAAGG |
| IAAL BamHI R | TATGGTACCTCAGTTTCGGCGGTCGATGATG |
