# Supplementary material for: Patterning of Leaf Vein Networks by Convergent Auxin Transport Pathways
Source: PLoS Genet. 2013 Feb 21;9(2):e1003294. doi: 10.1371/journal.pgen.1003294 (PMC3578778; doi:10.1371/journal.pgen.1003294)
Supplement: Table S4 — Imaging parameters: single-marker lines. (DOC) [file pgen.1003294.s007.doc]

**Table S4.** Imaging parameters: single-marker lines.

| **Line** | **Laser** | **Wavelength (nm)** | **Main dichroic beam splitter** | **First secondary dichroic beam splitter** | **Second secondary dichroic beam splitter** | **Emission filter (detector)** |
| --- | --- | --- | --- | --- | --- | --- |
| PIN6::PIN6:GFP | Ar | 488 | HFT 405/488/594 | NFT 545 | NFT 490 | BP 505-530 (PMT3) |
| PIN6::YFPnuc | Ar | 514 | HFT 405/514/594 | NFT 595 | NFT 515 | BP 520-555 IR (PMT3) |
| PIN8::PIN8:GFP | Ar | 488 | HFT 405/488/594 |  |  | 507-593 (META) |
| PIN8::YFPnuc | Ar | 514 | HFT 405/514/594 | NFT 595 | NFT 515 | BP 520-555 IR (PMT3) |
| DR5rev::YFPnuc | Ar | 514 | HFT 405/514/594 | NFT 595 | NFT 515 | BP 520-555 IR (PMT3) |
| PIN1::PIN1:YFP | Ar | 514 | HFT 405/514/594 | NFT 595 | NFT 515 | BP 520-555 IR (PMT3) |
| MP::YFPnuc | Ar | 514 | HFT 405/514/594 | NFT 595 | NFT 515 | BP 520-555 IR (PMT3) |
