# Supplementary material for: Patterning of Leaf Vein Networks by Convergent Auxin Transport Pathways
Source: PLoS Genet. 2013 Feb 21;9(2):e1003294. doi: 10.1371/journal.pgen.1003294 (PMC3578778; doi:10.1371/journal.pgen.1003294)
Supplement: Table S5 — Imaging parameters: multi-marker lines. (DOC) [file pgen.1003294.s008.doc]

**Table S5.** Imaging parameters: multi-marker lines.

| **Multi-marker lines** | **Single-marker lines** | **Laser** | **Wavelength (nm)** | **Main dichroic beam splitter** | **First secondary dichroic beam splitter** | **Second secondary dichroic beam splitter** | **Emission filter (detector)** |
| --- | --- | --- | --- | --- | --- | --- | --- |
| PIN6::PIN6:GFP; PIN6::YFPnuc; chlorophyll | PIN6::PIN6:GFP | Ar | 488 | HFT 405/488/594 | NFT 595 | NFT 545 | BP 505-530 (PMT2) |
|  | PIN6::YFPnuc | Ar | 514 | HFT 405/515/594 | NFT 595 | NFT 515 | BP 520-555 IR (PMT3) |
|  | chlorophyll | Ar | 488 | HFT 405/488/594 | NFT 595 |  | 604-700 (META) |
| PIN1::PIN1:CFP; PIN6::YFPnuc; chlorophyll | PIN1::PIN1:CFP | Ar | 458 | HFT 458/514 | NFT 595 | NFT 545 | BP 470-500 (PMT2) |
|  | PIN6::YFPnuc | Ar | 514 | HFT 458/514 | NFT 595 | NFT 545 | BP 530-575 IR (PMT3) |
|  | chlorophyll | Ar | 458 | HFT 458/514 | NFT 595 |  | 604-700 (META) |
| PIN6::PIN6:GFP; 35S::YFPer | PIN6::PIN6:GFP | Ar | 458 | HFT 458/514 | Mirror | NFT 515 | BP 470-500 (PMT2) |
|  | 35S::YFPer | Ar | 514 | HFT 458/514 | Mirror | NFT 515 | BP 575-620 IR (PMT3) |
| PIN6::PIN6:GFP; 35S::RTNLB4:YFP | PIN6::PIN6:GFP | Ar | 458 | HFT 458/514 | Mirror | NFT 545 | BP 475-525 (PMT2) |
|  | 35S::RTNLB4:YFP | Ar | 514 | HFT 458/514 | Mirror | NFT 515 | BP 530-575 IR (PMT3) |
| J1721::GFPer; 35S::YFPer | J1721::GFPer | Ar | 458 | HFT 458/514 | Mirror | NFT 515 | BP 470-500 (PMT2) |
|  | 35S::YFPer | Ar | 514 | HFT 458/514 | Mirror | NFT 515 | BP 575-620 IR (PMT3) |
| ATHB8::GFPnuc; 35S::YFPer | ATHB8::GFPnuc | Ar | 458 | HFT 458/514 | Mirror | NFT 515 | BP 470-500 (PMT2) |
|  | 35S::YFPer | Ar | 514 | HFT 458/514 | Mirror | NFT 515 | BP 575-620 IR (PMT3) |
| PIN6::PIN6:GFP; 35S::YFPpm | PIN6::PIN6:GFP | Ar | 458 | HFT 458/514 | Mirror | NFT 515 | BP 470-500 (PMT2) |
|  | 35S::YFPpm | Ar | 514 | HFT 458/514 | Mirror | NFT 515 | BP 575-620 IR (PMT3) |
| J1721::GFPer; 35S::YFPpm | J1721::GFPer | Ar | 458 | HFT 458/514 | Mirror | NFT 515 | BP 470-500 (PMT2) |
|  | 35S::YFPpm | Ar | 514 | HFT 458/514 | Mirror | NFT 515 | BP 575-620 IR (PMT3) |
| 35S::YFPpm; FM4-64 | 35S::YFPpm | Ar | 488 | HFT 488/543 | Mirror | NFT 545 | BP 505-530 (PMT2) |
|  | FM4-64 | HeNe | 543 | HFT 488/543 | Mirror | NFT 545 | BP 560-615 IR (PMT3) |
| PIN8::PIN8:GFP;  ER-Tracker Red | PIN8::PIN8:GFP | Ar | 488 | HFT 488/543 | Mirror | NFT 545 | BP 505-530 (PMT2) |
|  | ER-Tracker Red | HeNe | 543 | HFT 488/543 | Mirror | NFT 545 | BP 600-650 (PMT3) |
| ER-Tracker Blue-White DPX;  PIN8::PIN8:GFP | ER-Tracker Blue-White DPX | Diode | 405 | HFT 405/488/543 | Mirror | NFT 490 | BP 420-480 (PMT2) |
|  | PIN8::PIN8:GFP | Ar | 488 | HFT 405/488/543 | Mirror | NFT 490 | BP 505-530 (PMT3) |
| ATHB8::CFPnuc; PIN1::PIN1:YFP | ATHB8::CFPnuc | Ar | 458 | HFT 458/514 | NFT 595 | NFT 515 | BP 470-500 (PMT2) |
|  | PIN1::PIN1:YFP | Ar | 514 | HFT 458/514 | NFT 595 | NFT 515 | BP 520-555 IR (PMT3) |
